# Brazilian Society of Otology task force – cochlear implant ‒ recommendations based on strength of evidence

**DOI:** 10.1016/j.bjorl.2024.101512

**Published:** 2024-09-16

**Authors:** Robinson Koji Tsuji, Rogério Hamerschmidt, Joel Lavinsky, Felippe Felix, Vagner Antonio Rodrigues Silva

**Affiliations:** a Universidade de São Paulo (USP), Faculdade de Medicina, Departamento de Otorrinolaringologia, São Paulo, SP, Brazil; b Universidade Federal do Paraná (UFPR), Departamento de Otorrinolaringologia, Curitiba, PR, Brazil; c Universidade Federal do Rio Grande do Sul (UFRGS), Departamento de Ciências Morfológicas, Porto Alegre, RS, Brazil; d Universidade Federal do Rio de Janeiro (UFRJ), Hospital Universitário Clementino Fraga Filho (HUCFF), Rio de Janeiro, RJ, Brazil; e Universidade de Campinas (Unicamp), Faculdade de Ciências Médicas (FCM), Departamento de Otorrinolaringologia e Cirurgia de Cabeça e Pescoço, Campinas, SP, Brazil

**Keywords:** Hearing loss, sensorineural, Deafness, Cochlear implantation, Cochlear implant, Implantable hearing device

## Abstract

**Objective:**

To make evidence-based recommendations for the indications and complications of Cochlear Implant (CI) surgery in adults and children.

**Methods:**

Task force members were educated on knowledge synthesis methods, including electronic database search, review and selection of relevant citations, and critical appraisal of selected studies. Articles written in English or Portuguese on cochlear implantation were eligible for inclusion. The American College of Physicians’ guideline grading system and the American Thyroid Association’s guideline criteria were used for critical appraisal of evidence and recommendations for therapeutic interventions.

**Results:**

The topics were divided into 2 parts: (1) Evaluation of candidate patients and indications for CI surgery; (2) CI surgery – techniques and complications.

**Conclusions:**

CI is a safe device for auditory rehabilitation of patients with severe-to-profound hearing loss. In recent years, indications for unilateral hearing loss and vestibular schwannoma have been expanded, with encouraging results. However, for a successful surgery, commitment of family members and patients in the hearing rehabilitation process is essential.

## Highlights

• Hearing preservation in Cochlear Implant (CI) surgery should be encouraged.
• Even for patients with auditory nerve mutations, CI can still be recommended.
• Despite gene therapy in preliminary studies with OTOF gene, CI is still indicated.
• Patients with surgical wound infection should be evaluated and treated as quickly.

## Introduction

Cochlear Implant (CI) is the first implantable device that successfully stimulates one of the five senses. Until the availability of CIs, children with severe-to-profound bilateral hearing loss using Personal Sound Amplification Products (PSAPs) acquired oral language with difficulty or did not develop it, directly impacting their social life and school performance. CIs have made it possible for many children with severe-to-profound bilateral hearing loss to achieve age-appropriate expressive and receptive language skills by the time they enter elementary school.^1^ Adults with progressive or sudden hearing loss who cannot benefit from hearing aids also have excellent results with CIs, depending on the cause of the deafness.^2^

As computer and hearing aid technology has advanced, the components required to produce CI hardware and software have also evolved. The technology that goes into the hardware was only part of the necessary considerations for CI design. Manufacturers needed to ensure biocompatibility, owing to concerns about infections such as meningitis due to the presence of a foreign body in a space that communicates with the Cerebrospinal Fluid (CSF).^3^ These CI design considerations were challenging and took a significant amount of time to perfect.

The concept is similar among the manufacturers. The CI consists of an external device that receives and processes the sounds, transforms them into electromagnetic energy, and sends them to the internal device that directly stimulates the cochlear nerve. Electrode arrays are the component of CI that have evolved more than any other over the years. The single channel electrode has been replaced with the multiple channel electrode. The stiffness and flexibility of the electrode array have been improved to optimize insertion length and reduce trauma during insertion.

## Objective

To make evidence-based recommendations for the indications and complications of CI surgery in adults and children.

## Methods

The Brazilian Society of Otology (Sociedade Brasileira de Otologia, SBO) met some members to discuss the topic of this guideline. Each author was asked to write a text with the current literature on the topic, based on evidence and containing the elements discussed during the meeting. A rapporteur prepared the final text, which was reviewed by additional coauthors and the Brazilian Journal of Otorhinolaryngology editor.

This guideline is not intended to be a substitute for individual professional judgment. Physicians should always act and decide in a way that they believe is best for their patients, regardless of guideline recommendations. They should also operate within their scope of practice and in accordance with their training. The guidelines represent the best judgment of a team of experienced physicians addressing the scientific evidence for a given topic.

The grading system of the American College of Physicians (ACP) was used in this guideline, relating to critical appraisal and recommendations on therapeutic interventions (Table 1, Table 2).^4^ An important component of this guideline was judged to be critical appraisal of diagnostic testing studies. However, the ACP guideline grading system was not designed for this purpose.^5^, ^6^, ^7^

**Table 1:** Interpretation of the American College of Physicians’ Guideline Grading System (for Therapeutic Interventions).

Recommendation	Clarity of risk/benefit	Implications
Strong recommendation	Benefits clearly outweigh harms and burdens, or vice versa.	Patients: Most would want course of action; a person should request discussion if an intervention is not offered.
		Clinicians: Most patients should receive the recommended course of action.
		Policymakers: The recommendation can be adopted as policy in most circumstances.
Weak recommendation	Benefits closely balanced with harms and burdens.	Patients: Many would want course of action, but some may not; the decision may depend on individual circumstances.
		Clinicians: Different choices will be appropriate for different patients; the management decision should be consistent with patients’ preferences and circumstances.
		Policymakers: Policymaking will require careful consideration and stakeholder input.
No recommendation	Balance of benefits and risks cannot be determined.	Decisions based on evidence cannot be made.

**Table 2:** Recommendations (for Therapeutic Interventions) Based on Strength of Evidence.

Recommendation and evidence of quality	Description of supporting evidence^a^	Interpretation
Strong recommendation		
High-quality evidence	RCT without important limitations or overwhelming evidence from observational studies.	Can apply to most patients in most circumstances without reservation.
Moderate-quality evidence	RCT with important limitations or strong evidence from observational studies.	Can apply to most patients in most circumstances without reservation.
Low-quality evidence	Observational studies/case studies.	May change when higher-quality evidence becomes available.
Weak recommendation		
High-quality evidence	RCT without important limitations or overwhelming evidence from observational studies.	Best action may differ based on circumstances or patients’ values.
Moderate-quality evidence	RCT with important limitations or strong evidence from observational studies.	Best action may differ based on circumstances or patients’ values.
Low-quality evidence	Observational studies/case studies.	Other alternatives may be equally Reasonable.
Insufficient	Evidence is conflicting, of poor quality, or lacking.	Insufficient evidence to recommend for or against.

RCT, Multicenter Randomized Controlled Trial.

a This description of supporting evidence refers to therapy, therapeutic strategy, or prevention studies. The description of supporting evidence is different for diagnostic accuracy studies.

The American Thyroid Association (ATA) created a diagnostic test appraisal system that used the following methodological elements: consecutive recruitment of patients representative of clinical practice, use of an appropriate reference gold standard, directness of evidence (target population of interest, testing procedures representative of clinical practice, and relevant outcomes), precision of diagnostic accuracy measures (confidence intervals for estimates such as sensitivity and specificity), and consistency of results across studies using the same test that was also used in this guideline (Table 3, Table 4).^6^

**Table 3:** Interpretation of the American Thyroid Association Guideline for Diagnostic Tests.

Recommendation	Accuracy of diagnostic information vs. risks and burden of testing	Implications
Strong recommendation	Knowledge of the diagnostic test result clearly outweighs risks and burden of testing or vice versa.	Patients: In the case of an accurate test for which benefits outweigh risks/burden, most would want the diagnostic to be offered (with appropriate counseling). A patient should request discussion of the test if it is not offered. In contrast, for a test in which risks and burden outweigh the benefits, most patients should not expect the test to be offered.
		Clinicians: In the case of an accurate test for which benefits outweigh risks/burden, most patients should be offered the diagnostic test (and provided relevant counseling). Counseling about the test should include a discussion of the risks, benefits, and uncertainties related to testing (as applicable), as well as the implications of the test result. In contrast, for a test in which risks and burden outweigh the perceived benefits, most patients should not be offered the test, or if the test is discussed, the rationale against the test should, for the particular clinical situation, be explained.
		Policymakers: In the case of an accurate test for which benefits outweigh risks/burden, availability of the diagnostic test should be adopted in health policy. In contrast, for a test in which risks and burden outweigh the perceived benefits, some restrictions on circumstances for test use may need to be considered.
Weak recommendation	Knowledge of the diagnostic test result is closely balanced with risks and burden of testing.	Patients: Most would want to be informed about the diagnostic test, but some would not want to seriously consider undergoing the test; a decision may depend on the individual circumstances (e.g., risk of disease, comorbidities, or other), the practice environment, feasibility of optimal execution of the test, and consideration of other available options.
		Clinicians: Different choices will be appropriate for different patients, and counseling about the test (if being considered) should include a discussion of the risks, benefits, and uncertainties related to testing (as applicable), as well as the implications of the test result. The decision to perform the test should include consideration of the patients’ values, preferences, feasibility, and the specific circumstances. Counseling the patient on why the test may be helpful or not, in her/his specific circumstance, may be highly valuable in the decision-making process.
		Policymakers: Policymaking decisions on the availability of the test will require discussion and stakeholder involvement.
No recommendation	Balance of knowledge of the diagnostic test result cannot be determined.	Decisions on the use of the test based on evidence from scientific studies cannot be made.

**Table 4:** Recommendations (for diagnostic interventions) based on strength of evidence.

Recommendation and evidence of quality	Methodological quality of supporting evidence	Interpretation
Strong recommendation		
High-quality evidence	Evidence from one or more well-designed nonrandomized diagnostic accuracy studies (i.e., observational ‒ cross-sectional or cohort) or systematic reviews/meta-analyses of such observational studies (with no concern about internal validity or external generalizability of the results).	Implies the test can be offered to most patients in most applicable circumstances.
Moderate-quality evidence	Evidence from nonrandomized diagnostic accuracy studies (cross-sectional or cohort), with one or more possible limitations causing minor concern about internal validity or external generalizability of the results.	Implies the test can be offered to most patients in most applicable circumstances without reservation.
Low-quality evidence	Evidence from nonrandomized diagnostic accuracy studies with one or more important limitations causing serious concern about internal validity or external generalizability of the results.	Implies the test can be offered to most patients in most applicable circumstances, but the utilization of the test may change when higher-quality evidence becomes available.
Weak recommendation		
High-quality evidence	Evidence from one or more well-designed nonrandomized diagnostic accuracy studies (i.e., observational ‒ cross-sectional or cohort) or systematic reviews/meta-analyses of such observational studies (with no concern about internal validity or external generalizability of the results).	The degree to which the diagnostic test is seriously considered may differ depending on circumstances or patients’ or societal values.
Moderate-quality evidence	Evidence from nonrandomized diagnostic accuracy studies (cross-sectional or cohort), with one or more possible limitations causing minor concern about internal validity or external generalizability of the results.	The degree to which the diagnostic test is seriously considered may differ depending on individual patients’/practice circumstances or patients’ or societal values.
Low-quality evidence	Evidence from nonrandomized diagnostic accuracy studies with one or more important limitations causing serious concern about internal validity or external generalizability of the results.	Alternative options may be equally reasonable.
Insufficient	Evidence may be of such poor quality, conflicting, lacking (i.e., studies not done), or not externally generalizable to the target clinical population such that the estimate of the true effect of the test is uncertain and does not permit a reasonable conclusion to be made.	Insufficient evidence exists to recommend for or against routinely offering the diagnostic test.

## Results

### Evaluation of candidate patients and indications for cochlear implant

Indications for CI surgery should be considered carefully and involve a number of factors before the procedure can be performed.^8^, ^9^

• Patient and family goals and expectations – It is crucial to understand what the family and patient expect from the procedure and align expectations with clinical reality.
• Family involvement – Family support and involvement are critical to the success of CI, especially in children, ensuring consistent device use and active participation in speech therapy.
• Consistent device use – The CI should be used for as long as possible each day to obtain the best results.
• Realistic expectations – Although CI can significantly improve hearing, it is important that families and patients have realistic expectations. Progress may be slow, and patients will need ongoing support to develop their listening and speaking skills.
• Prognosis assessment – The etiology of hearing loss, patient age, duration of auditory deprivation and the presence of other pathologies should be considered to estimate good or bad prognosis.

The indication for CI surgery requires coordination between otolaryngologists, speech therapists, audiologists, psychologists, and other health professionals, such as neurologists, pediatricians, radiologists, psychiatrists, social workers, and educators, varying according to the needs of each patient and the reality of the auditory rehabilitation center. The criteria to indicate CI are not uniform throughout the world.

The clinical visit should include a precise and comprehensive history-taking to support not only the diagnosis but also the prognosis regarding the progression of hearing loss, indications for CI, expectations for post-implant development, and counseling. The main questions that should be considered are as follows: (1) Onset and diagnosis of hearing impairment; (2) History of hospitalization and comorbidities; (3) Family history of hearing impairment; (4) Pregnancy history (mainly in children); (5) Perinatal history; and (6) Length of time using hearing aids (including current use or no use).

The worst postoperative outcomes are associated with increased duration of deafness (auditory deprivation), inconsistent use of hearing aids, presence of syndromic causes, perinatal problems, cochlear ossification, and atypical cochlear anatomy – especially changes in the anatomy of the Internal Auditory Canal (IAC) and Common Cavity (CC) deformity.^1^, ^10^

Physicians evaluating patients who are candidates for CI should consider family and personal history. Family history allows a detailed assessment of the presence of premature, severe-to-profound, syndromic or nonsyndromic hearing loss. Medical history includes perinatal history (Cytomegalovirus [CMV] infection, prematurity, kernicterus, hypoxia), diabetes mellitus, hypertension, and uncontrolled arrhythmia, as well as otologic history (noise exposure, ototoxic drugs, chronic ear disease, ear surgery, trauma).

### Hearing assessment

In addition to a detailed history and physical examination, audiologic evaluation is the first step in the investigation. The following tests are typically available:

a) Pure-tone and speech audiometry;
b) Transient-Evoked Otoacoustic Emissions (TEOAEs) and Distortion-Product Otoacoustic Emissions (DPOAEs);
c) Auditory Brainstem Response (ABR);
d) Tone-Burst ABR (TBABR) or Auditory Steady-State Response (ASSR);
e) Free-field audiometry.

#### Pure-tone and speech audiometry

Audiometric thresholds are the gold standard for the diagnosis and assessment of hearing thresholds. However, pure-tone audiometry requires consistent responses from patients and its reliability depends on their attention and cooperation, which may be difficult to obtain at certain ages or health conditions.^11^, ^12^ Hearing thresholds can be objectively determined using electrophysiologic tests, such as ABR, TBABR, ASSR, and cortical auditory evoked potentials.

Word recognition tests and sentence recognition tests, or a mixture of both, are primarily used to assess speech discrimination.^13^ Protocols for incorporating noise into CI evaluation vary from institution to institution. However, patients with an indication for surgery based on noise tests have been shown to benefit from CI.^14^ Monosyllabic word recognition is an alternative tool due to performance ceiling effects and the semantic context associated with sentences.^15^ Monosyllables are challenging and provide a more accurate metric for improving patient monitoring.

Sladen et al.^15^ evaluated the effect of CI in patients with consonant-nucleus-consonant monosyllabic word recognition scores of up to 40% and found that even when consonant-nucleus-consonant word scores only reached 80%, CI still provided benefits. Furthermore, other studies have also shown positive results in patients whose performance exceeded “traditional” criteria.^16^, ^17^, ^18^ They suggested a reassessment of candidacy criteria to include patients who did not benefit from conventional amplification, considering word recognition a key indicator of the potential benefit of CI.

#### Otoacoustic emissions and auditory brainstem response

Although the “1-3-6 plan” (screening infants for hearing loss by 1-month of age, having a diagnostic audiologic evaluation by 3-months of age, and enrolling in appropriate intervention by 6-months of age)^19^ assists in establishing timely audiologic intervention in children, it is essential to define which modality of hearing rehabilitation these children should receive to prevent auditory deprivation and ensure maximum exposure to auditory information during the sensitive period of the 4 speech and language development domains – speech production, speech perception, receptive language, and auditory performance.^20^

Children with profound congenital deafness will not benefit from hearing amplification; therefore, intervention requires the adoption of sign language, CI, or often a combination of both. In a 2019 update, the Joint Committee on Infant Hearing encouraged the programs that met the 1-3-6 benchmark to meet a 1-2-3 month timeline for screening, diagnosis, and intervention, respectively.^21^

Newborn Hearing Screening (NHS) should be done up to 1-month of life, diagnosis of hearing loss be made up to 2-months of age, and rehabilitation be started up to 3-months of age.^21^ Early intervention with a hearing aid or CI is critical to successful rehabilitation outcomes. The test most commonly used for NHS is TEOAEs, in patients without risk factors. OAEs can rapidly measure middle ear and cochlear function. If present, the middle ear or cochlea (Outer Hair Cells [OHCs]) are highly likely to be normal. The most common tests used to diagnose hearing loss are TEOAEs and DPOAEs.

TEOAEs represent the sum of OHC pulse responses along the cochlea, whereas DPOAEs arise directly from the frequency-selective compressive nonlinearity of OHC amplifiers. In principle, both DPOAEs and TEOAEs allow acquisition of frequency-specific information about a hearing loss problem.^22^

When stimulating the ear with a transient stimulus, almost all OHCs along the cochlea (when using a click) or a part of them (when using a tone-burst) are stimulated. Because of frequency dispersion in the cochlea, a specific component of the TEOAE response can be directly attributed to a specific frequency component of the transient signal. Because the basilar membrane at basal sites moves faster than at more apical sites, high-frequency TEOAE components come from basal cochlear sites, whereas low-frequency TEOAE components come from more apical sites. However, because the stimulus and the high-frequency TEOAE components overlap (and therefore have to be canceled during TEOAE recording), TEOAEs cannot measure cochlear function above 4 kHz. In contrast, DPOAEs are able to assess OHC function at different sites of the cochlea separately depending on the primary-tone frequency.^22^

There are some limitations when performing OAE measurements. Electric microphone noise, physiologic noise (breathing, blood flow), and external acoustic noise do not allow reliable measurements below 1 kHz. Because of the limited frequency range of the sound probe’s electroacoustic transducers, high-frequency OAE measurements are difficult without the use of specialized devices.^22^, ^23^

OAEs are a fast measure to confirm normal middle ear and cochlear function. This is the case if OAEs are present over a wide frequency range. In the absence of OAEs, middle ear or cochlear (OHC) pathology is likely. OAEs should then be followed by tympanometry. If the tympanogram is abnormal, conductive hearing loss is likely. If the tympanogram is normal and OAEs are abnormal or absent, a cochlear disorder is likely. If both tympanogram and OAEs are normal, and hearing impairment is still suspected, ABRs are needed to assess whether there is a cochlear (Inner Hair Cell [IHC]) or neural pathology.^22^

Especially for hearing aid fitting in infants, quantitative assessment of hearing loss is necessary. When acquired by high stimulus levels (which is common in clinical practice), TEOAEs are absent at a cochlear hearing loss exceeding 20 dB HL, whereas DPOAEs are absent only at a cochlear hearing loss exceeding 40–50 dB HL. Thus, when TEOAEs and DPOAEs are used at high stimulus levels, it is possible to roughly estimate hearing loss. For example, when TEOAEs are absent and DPOAEs are present, hearing loss is suggested not to exceed 30 dB HL.^24^

A reliable diagnosis of hearing impairment in newborns referred from NHS is only ensured if as many objective audiometric tests as possible are performed: tympanometry to assess middle ear status, OAEs to assess the function of cochlear amplification, and ABRs to assess synaptic and neural functionality. TEOAEs assess cochlear function more qualitatively and are therefore more suitable for diagnosis.^11^, ^12^

DPOAE audiograms are able to assess cochlear hearing loss more accurately than behavioral tests in infants where the conditioned free-field audiogram does not reflect the real threshold. Additionally, unilateral hearing loss can be detected. DPOAE audiograms can reveal transient conductive hearing loss in the early postnatal period and are an alternative method to TBABR and ASSR in cases of mild or moderate hearing loss. Test time to establish a DPOAE audiogram takes only a few minutes. Thus, DPOAE audiograms have an essential advantage over ABRs and can help establish a working hypothesis for a more successful hearing aid fitting.^22^, ^25^

A two-stage NHS should be conducted. Children who “failed” the first evaluation with TEOAE, which is more sensitive for diagnosing hearing loss than DPOAE, should undergo a retest that can be the same as before or another method. It is important that this retest is performed before 1 month of life, preferably before discharge from the maternity hospital if possible, and that there is no diagnostic delay in case of new “failed” retests. If the first and second evaluations fail, even in one of the ears, the child should be immediately referred for otolaryngologic evaluation and diagnostic testing.^25^

NHS may not identify mild losses due to TEOAE limitations (less than 25–40 dB) more often when performed with automated ABR,^25^ while detection of auditory neuropathy may fail especially if screening is performed with TEOAE.^26^ In addition, late or progressive losses may not be detected, which makes follow-up with hearing and language developmental milestones essential. Regardless of NHS outcomes, all infants and children should be routinely monitored for hearing, cognitive development, communication, achievement of educational milestones, general health, and well-being.^27^

TBABR and ASSR can provide estimates of the type and degree of hearing loss by performing air- and bone-conduction testing. These results facilitate hearing aid fitting at an early age.^28^ There is a strong correlation between click ABR and TBABR/ASSR thresholds and subsequent behavioral hearing thresholds from 1000 to 4000 Hz.^29^ Even when children produce no response on diagnostic ABR testing, hearing thresholds should be confirmed with behavioral testing.^11^

#### Speech assessment

The indication for CI surgery also depends on an adequate speech assessment, considering patient age, ability to cooperate with the examination, and comorbidities. CI candidates have speech and/or language deficits due to insufficient access to appropriately fitted and consistently used hearing aids. Speech and language testing should be performed by experienced professionals to allow for a comprehensive discussion of expectations and appropriate goal setting. Audiologists and speech therapists should also discuss the spectrum of communication options, which range from reliance on sign language to spoken language.

Speech therapists are essential for the indication and success of CI surgery, in addition to adjusting the expectations of family members and CI candidates and evaluating the individual’s motivation to listen. A detailed assessment should be conducted, including history-taking, evaluation of established communication, patient language, speech, and voice, as well as auditory performance and speech perception with and without PSAPs. Pediatric audiologists should perform behavioral testing during CI evaluation. It is important to obtain specific information necessary for device selection – ear to be implanted and consideration of bimodal implantation.^30^

Minimum hearing experience is essential for most candidates, even for those who report no benefit from the use of PSAPs. Any experience with acoustic cues, even with suprasegmental traces, will favor the activation of auditory pathways. It is important that patients have an adequate level of communication, whether by lip reading or sign language, as they will receive much information and guidance that should be clearly understood and apprehended.

This discussion may require multiple sessions from multiple CI team members to ensure parental understanding and informed choice of communication options. The CI team should value patient and family choices. Furthermore, in the case of pediatric patients, depending on their age in relation to the critical windows for auditory and language development, using a CI does not guarantee fluent use of spoken language, but this does not negate other potential benefits, such as increased environmental sound awareness and general well-being. Regardless of the mode of communication, counseling should include discussions with the family about the use of appropriate amplification during all waking hours and realistic expectations of how a CI can benefit the child.

Higher socioeconomic status may affect a child’s age at implantation, speech, language, and hearing outcomes.^31^ The family’s financial and support resources do not override the decision to undergo surgery but may highlight the need for additional support and resources. Child and family commitment is crucial to the success of surgery and language acquisition. Family involvement contributes significantly to the development of language skills in children with hearing loss.^8^

Children using CIs for at least 10 h a day learn language faster than those with less daily device use.^32^ There is evidence showing the link between mean daily CI use and early auditory skills, speech recognition, and language skills,^33^, ^34^ but there is a lack of good evidence to recommend a minimum daily PSAP use for children before surgery. Park et al.^35^ reported better receptive and expressive language in children using PSAPs for at least 80% of age-appropriate “hearing hours” (accounting for differences in sleep patterns by age) before CI. Most children with hearing impairment have some unaided or aided access to sound and speech. Therefore, therapy should begin soon after the hearing loss is identified, and hearing aids are received. Therapy while waiting for a CI allows for early development of auditory skills, functional listening, speech, and language skills to the best of the patient’s ability. Involving parents/caregivers and family members in therapy sessions prepares them to assist in the ongoing development of communication skills.

#### Assessment by social workers, psychologists, and other health professionals

The inclusion of social workers, psychologists, and educators can improve the assessment of the child’s cognitive function and overall development, in addition to evaluating the family’s support, commitment, and motivation. These health professionals can engage in the follow-up appointments and hearing (re)habilitation protocol required to maximize post-implantation outcomes.^36^, ^37^ Recognizing and managing potential barriers to family participation also underlie a child’s success after implantation. A social worker could help facilitate accommodations, such as by arranging transportation or daycare, assisting with necessary documentation, or coordinating appointments.

A psychologist’s evaluation of a patient’s level of functioning and mental well-being can direct family-based discussions about realistic expectations.^38^ First, nonverbal intelligence consistently predicts communication outcomes with a CI,^39^, ^40^ although these relationships may be confounded by language skills. Second, assessment of psychological factors (e.g., emotions, internalizing and externalizing behaviors) may benefit a candidacy evaluation due to higher rates of aggression, anxiety, and attention deficits, particularly in children with hearing impairment.^41^

#### Recommendations

I Pure-tone and speech audiometry are the gold standard for hearing assessment and should be indicated for patients with adequate conditions to undergo them. (Strong Recommendation – High-Quality Evidence)
II Hearing assessment to indicate CI in children and adults requires objective tests such as OAEs and ABR. (Strong Recommendation – High-Quality Evidence).
III TBABR or ASSR are essential for CI surgery, especially in children and infants who do not have speech or pure-tone thresholds. (Strong Recommendation – Moderate-Quality Evidence).
IV Protocols for incorporating noise into CI evaluations can be used, especially for patients with preserved low-frequency hearing but poor outcomes with hearing aids. (Weak Recommendation – Moderate-Quality Evidence).
V Tympanometry may be useful, especially in young children, to distinguish between conductive hearing losses. But in children under 6-months of age, it should be performed with a 1000 Hz probe. (Weak Recommendation – Moderate-Quality Evidence).
VI Hearing loss in children should be diagnosed as early as possible to initiate appropriate treatment and provide the best language development possible. (Strong Recommendation – High-Quality Evidence).
VII Children with a normal neonatal screening should be routinely monitored for hearing, cognitive development, communication, and school performance. (Strong Recommendation – Moderate-Quality Evidence).
VIII Speech therapy evaluation is essential for indicating CI surgery and should be conducted by an experienced professional. (Strong Recommendation – Moderate-Quality Evidence).
IX The inclusion of social workers, psychologists, and educators should be considered and encouraged to assist in the assessment of cognitive function, patient development, and real motivation to undergo CI surgery. (Strong Recommendation – Moderate-Quality Evidence).

### Laboratory testing

Laboratory tests are not necessary for etiologic investigation without any clinical suspicion due to the low diagnostic yield of these tests (0%–2%).^42^ There is a discussion in the literature about the indication of universal NHS for congenital CMV infection. This is a highly prevalent virus, and diagnosis is only possible if the infection is detected within the first 30 days of life. But it is still not performed routinely.^43^

Genetic testing should be considered for CI candidates, especially children. It has a high yield, detecting the condition in 44% of patients with bilateral sensorineural hearing loss.^44^ GJB2 gene mutations account for more than 50% of cases of sensorineural hearing loss due to autosomal recessive inheritance and 20% of cases of prelingual hearing loss in high-income countries.^45^ One of the most common GJB2 mutations is 35delG, accounting for 70% of cases.^46^, ^47^ Therefore, it should be considered in children. Other mutations such as those in the SLC26A4 gene are less frequent.^48^

The investigation may be complemented by a renal ultrasound to check for congenital malformations, an electrocardiogram to rule out long QT syndrome (as seen in Jervell and Lange-Nielsen syndrome), and other tests based on clinical findings.

### Imaging

Imaging plays a role in determining the etiology, identifying abnormalities related to hearing loss progression, and surgical planning.^49^, ^50^ It allows the evaluation of the anatomy of the structures of the cochlea, labyrinth, cranial nerves, and central auditory pathways. Some inner ear malformations may contraindicate CI surgery.^51^

The diagnostic yield of imaging studies in the evaluation of children with profound bilateral sensorineural hearing loss ranges from 7% to 74%, with high specificity and high positive predictive value.^52^ The choice of imaging modality should take into account radiation exposure, diagnostic accuracy, duration, need for sedation, and cost.^53^

Imaging modalities for the assessment of sensorineural hearing loss include Computed Tomography (CT) and Magnetic Resonance Imaging (MRI). CT provides better visualization of bone structures, lower costs, and faster imaging, which makes it easier to use. However, CT scans expose patients to ionizing radiation, and longitudinal studies have shown an increased lifetime risk of developing certain types of cancer in children.^54^ MRI provides better quality images of soft tissues and neural structures, but it is associated with higher cost and longer acquisition time, often requiring sedation to be accomplished in children.^55^, ^56^

As an advantage, MRI can clearly identify the cochlear nerve on T2-weighted sequences, and abnormalities or tumors detected on MRI contraindicate CI. Concern has been raised about the role of gadolinium-based contrast agents in MRI due to controversy over gadolinium deposition in the brain.^57^ Thus, many recent pediatric hearing loss investigation protocols do not require gadolinium. In adults with postlingual deafness, CT has been advocated if there is concern for chronic otitis media or a history of middle ear disease, whereas MRI would be essential in patients with a history of meningitis or asymmetric sensorineural hearing loss.^58^, ^59^

There is considerable variation between clinical practice and institutional protocols in diagnostic methods used.^42^, ^60^ There is a correlation between diagnostic yield and hearing loss severity, with an increase of almost 20% in diagnostic yield in severe-to-profound hearing loss compared with mild-to-moderate hearing loss.^61^ The diagnostic yield is lower in patients who test positive for mutations in the GJB2 gene, which is the most common cause of nonsyndromic hearing loss.^62^

Two systematic reviews indicate that CT appears to have a higher diagnostic yield than MRI to diagnose Enlarged Vestibular Aqueduct (EVA) and cochlear abnormalities, whereas MRI can detect more cochlear nerve abnormalities.^63^, ^64^ In a multicenter study evaluating the diagnostic yield of CT vs MRI in children with profound bilateral hearing loss, the concordance rate between CT and MRI was 92%, with the diagnostic yield of CT being 7% higher than that of MRI (*p* = 0.0001).^65^ In another study with a similar design comparing diagnostic yield between CT and MRI, the concordance rate between them was 86%, with MRI having a 14% higher yield for detecting diagnostic findings in children with profound bilateral sensorineural hearing loss.^66^

In cases of auditory neuropathy, MRI proved to be highly effective at detecting cochlear nerve hypoplasia, a common finding in these cases.^67^ Between 12% and 18% of children with congenital sensorineural hearing loss have hypoplasia or aplasia of the eighth cranial nerve. MRI has the highest diagnostic yield in these cases.^68^ The IAC may also be hypoplastic in these cases, which can be detected on CT.

The facial nerve has an abnormal course in 16% of children with inner ear malformations, representing increased difficulty in CI surgery, especially when it runs across the promontory or makes the facial recess narrower. In some children, the facial nerve may be split, and a second nerve may be located anteriorly, in addition to a branch located in the normal position of the facial recess.^69^ In these cases, a detailed study with MRI and CT is recommended.

The most common inner ear malformation is EVA, which can be detected by CT or MRI. The modern radiologic definition of EVA has been established as a mean diameter greater than 1.5 mm.^69^ Mutations in the SLC26A4 gene are a common cause of EVA. It often accompanies other inner ear anomalies, such as incomplete partition type II. CT is the best imaging modality to assess the vestibular aqueduct. MRI offers complementary visualization of the endolymphatic sac and duct that are usually also enlarged.^48^

Hearing loss after meningitis is the most common cause of acquired bilateral sensorineural hearing loss in the pediatric population. During the acute stage, the CT scan may be normal, but MRI reveals intense cochlear enhancement,^70^ and it is important to use contrast in these cases. In later stages, fibrosis is shown by loss of T2 signal in the cochlea. Cochlear ossification can be seen on both CT and MRI. MRI is essential to assess the extent of injury and feasibility of CI surgery in these cases.

Regardless of the choice of imaging modality, visualization of the cochlea is essential before CI. Although imaging often has little influence on the approach for CI surgery in adults with postlingual deafness, it provides necessary insights in the evaluation of hearing impairment in children that can have a major impact on CI decision-making.

Traumatic brain injury can lead to drastic conditions, such as facial paralysis and profound sensorineural hearing loss with involvement of the otic capsule. CT is essential in these cases to properly see the fracture lines. Longitudinal fractures, generally sparing the otic capsule, account for the majority of temporal bone fractures. In transverse fractures, due to the direction of the fracture line toward the otic capsule, patients often have some complications such as CSF leak, damage to the otic capsule, and injury to the facial nerve.^71^, ^72^

#### Recommendations

X It is not recommended to routinely perform laboratory tests, without clinical suspicion, for the etiologic diagnosis of patients with an indication for CI. (No recommendation).
XI Polymerase chain reaction testing to diagnose congenital CMV infection should only be performed in the first month of life, and serology is not recommended. (Strong Recommendation – Moderate-Quality Evidence).
XII Genetic testing is recommended for children with profound bilateral congenital hearing loss due to the high diagnostic yield, especially in the search for GJB2 gene mutations. (Strong Recommendation – High-Quality Evidence).
XIII Imaging is recommended for CI candidates for diagnosis, planning, and even contraindication to CI surgery. (Strong Recommendation – High-Quality Evidence).
XIV MRI is mainly indicated in suspected cochlear ossification (deafness caused mainly by bacterial meningitis and otosclerosis) and cochlear nerve aplasia. (Strong Recommendation – Moderate-Quality Evidence).
XV CT is mainly indicated in patients with chronic otitis media and inner ear malformations due to a major risk of affecting the anatomy of the facial nerve. (Strong Recommendation – Moderate-Quality Evidence).

### Vestibular assessment

The vestibular system consists of 3 semicircular canals (anterior, posterior, and lateral) and 2 otolith organs (saccule and utricle). Each of these 5 structures has sensory receptors consisting of highly specialized hair cells, with the ampullary cristae located in the ampullae of the semicircular canals, and the utricular and saccular maculae located in the respective otolith organs. The ampullary cristae are responsible for detecting angular accelerations, such as moving the head in different directions, whereas the maculae are responsible for detecting linear accelerations and head tilt, both in the vertical plane (saccular macula) and in the horizontal plane and in inclination (utricular macula).^73^, ^74^

Body balance in humans depends on the integration of multiple systems and organs that send information to the central nervous system, which processes this information and sends signals and reflexes to regulate posture and gait. Among these multiple sensory systems, the following stand out: vision, vestibular system, and proprioception (a bundle of nerves, muscles, and joints).^74^ The vestibular system has a close anatomic relationship with the cochlea, sharing the same bone scaffold and membranous ducts, having similar sensory hair cells, and sharing the same embryonic origin. Thus, disturbances in one system can affect the other system.^74^, ^75^

CIs can damage several vestibular organs, resulting in poor vestibular function, dizziness, and balance disorders.^76^ Multiple mechanisms contribute to vestibular dysfunction during or after surgery: (1) Direct trauma to the basilar membrane, modiolus, and spiral ligament from electrode insertion; (2) Acute serous labyrinthitis due to cochleostomy; (3) Foreign body reaction causing an inflammatory process, fibrosis, and osteogenesis; (4) Endolymphatic hydrops; and (5) Electrical stimulation of the labyrinth by the implant.^77^, ^78^

Most studies investigating vestibular function in patients before and after CI surgery have been conducted in adults because of the technical difficulties associated with performing vestibular tests in children. Some studies have reported an incidence of vertigo after CI surgery in adults ranging from 2% to 35%, whereas vestibular dysfunction ranges from 20% to 80% after surgery, depending on the method used to assess labyrinthine function.^79^

The literature has shown a close relationship and high prevalence of vestibular disorders in children with congenital sensorineural hearing loss, ranging from 20% to 70%.^80^, ^81^ Some studies even suggest a relationship between the etiology and severity of hearing loss and vestibular disorders. Children with severe-to-profound bilateral hearing loss would be more likely to have vestibular dysfunction, and some etiologies, such as Usher syndrome, congenital CMV infection, and GJB2 gene mutations, would also be more closely related to vestibular deficits.^81^, ^82^, ^83^

#### Preoperative vestibular assessment

Preoperative vestibular assessment helps detect pre-existing vestibular disorders and their potential impact on the postoperative period of CI surgery and subsequent rehabilitation needs.^84^ It establishes the patient’s baseline vestibular function, which serves as a reference point for comparison of postoperative changes.^85^ When unilateral surgery is indicated, it can be crucial in selecting the candidate ear for surgery, especially in a situation in which one ear has superior vestibular function.^86^ The results of vestibular assessment may provide support for considering a specific electrode design or surgical technique that can mitigate the impact on the vestibular system.^86^ However, they seem to be neglected by surgeons in most cases, especially in children, perhaps due to lack of knowledge of the importance and function of the vestibular system and/or the unavailability of diagnostic methods for pediatric vestibular assessment.^87^

Among the various clinically available vestibular assessment methods, all have some limitations. No method can assess all vestibular organs at once. Another major limitation of tests of labyrinthine function is that they evaluate reflex responses generated by the vestibular system, after a stimulus, and not the labyrinth directly. Several vestibular tests may be performed as part of the preoperative assessment:

1) Videonystagmography: It monitors eye movements in reaction to visual stimuli and head movements.
2) Caloric test: It assesses the function of the lateral semicircular canal in the low frequency spectrum. In most cases, the protocol includes 4 irrigations at different temperatures in both ears. Traditionally, it is a longer test and somewhat uncomfortable for most patients.^88^
3) Rotary chair testing: Patients sit in a motorized chair that rotates at varying speeds. It assesses the function of the semicircular canals and the Vestibulo-Ocular Reflex (VOR).
4) Vestibular Evoked Myogenic Potentials (VEMPs): There are 2 types of VEMPs, cervical VEMP (cVEMP) and ocular VEMP (oVEMP). These tests assess the function of otolith organs (saccule and utricle, respectively). The tests are of vestibular origin, and measurements are most commonly made in the sternocleidomastoid muscle (cVEMP) and in the inferior oblique extraocular muscle (oVEMP).
5) Clinical Head Impulse Test (cHIT): It is one of the most effective bedside tests to assess vestibular function, although not quantitatively. Despite being a simple test with high specificity that can be easily performed by any minimally trained health professional, there are some disadvantages ‒ it has an approximately 50% sensitivity, is examiner dependent, and provides only qualitative (not quantitative) information; that is, it does not accurately measure vestibular function.^89^
6) Video Head Impulse Test (vHIT): It is an evolution of the bedside cHIT. The vHIT is the only commercially available vestibular assessment method that can evaluate VOR gain in the 3 semicircular canals in the high frequency spectrum, in addition to being able to quantify the level of residual vestibular function.^89^ The goggle placement is very limited in children under 2-years of age. There are no models available with goggles that fit on young children’s faces.^90^
7) Posturography: Computerized dynamic posturography uses a force platform that creates instability. When combined with visual stimuli, it can be used to determine the relative importance of various sensory inputs critical to balance, vision, and somatosensory and vestibular systems.

Patient cooperation is mandatory and necessary for a few minutes. The cVEMP can be more easily performed, with electrodes being placed on the sternocleidomastoid muscle, forehead, and suprasternal notch. Because the test requires patient cooperation to be performed, most authors consider the cVEMP abnormal if there is no response in at least 3 consecutive attempts. Like vHIT, cVEMP is often used for vestibular assessment in children with hearing loss, and it can be performed successfully in most cases. The cVEMP is better tolerated than the oVEMP in this specific population.^91^ The rotary chair test has also been used in children.

Ibrahim et al.^92^ performed a meta-analysis of studies evaluating vestibular function or postural stability before and after CI surgery in patients aged ≥18 years. The results showed that CI surgery significantly affected the results of caloric and VEMP tests. However, the results of HIT, posturography, and dizziness handicap inventory (DHI) were not significantly affected after CI surgery. The meta-analysis data showed no significant increase in DHI scores in the majority of patients (84.4%), suggesting that CI did not affect patients’ balance perception. Several factors can contribute to the variability of results between the vestibular function tests, both before and after CI surgery, which are difficult to control for. These factors include age and etiology of hearing loss, surgical technique used, and incidence of trauma to the inner ear. However, the authors concluded that the possible effects of CI surgery on the vestibular system should be communicated to patients before CI surgery.

Pathologic studies of adult CI recipients have provided increased evidence of endolymphatic hydrops and saccular collapse. Probably for this reason, the cVEMP test appears to be statistically significantly altered in some studies when comparing results before and after surgery.^76^, ^79^ Interestingly, vHIT results before and after surgery have not shown the same abnormality rates of cVEMP and caloric test results, suggesting that the otolith organs are more vulnerable to damage caused by the surgical procedure than the semicircular canals, a finding compatible with the results of pathologic studies, and that high-frequency labyrinthine analysis methods are less sensitive than low-frequency methods for postoperative analysis.^79^

Vestibular dysfunction in childhood presents as a delay in some motor development milestones, such as neck control, sitting, crawling, and walking. This makes some children more susceptible to falls and deficits in fine motor skills, thus leading them to avoid, often unconsciously, exposure to more challenging situations involving body balance, such as walking on unstable surfaces (e.g., soft sand). Clearly marked signs, such as the presence of spontaneous nystagmus, are not so common because most patients have bilateral vestibular dysfunction.^90^

Performing vestibular tests in children is challenging, although approximately 50% of the children undergoing such tests are able to successfully complete a test battery.^93^ Among the various vestibular tests, cHIT and vHIT are the best tolerated by children (65.7%–94.2%).^91^ Therefore, it is suggested that cHIT be performed as a type of vestibular function screening in the pediatric population, as it is the best tolerated test, also considering that, despite its low sensitivity, a normal response is indicative of a likely normal VOR gain at high frequencies. In cases of uncertain cHIT results, or in the presence of a clearly abnormal response, the child may undergo other tests, with the vHIT, cVEMP, and caloric test being the most recommended ones (in this order).^90^

#### Recommendations

XVI There is a relationship between sensorineural hearing loss and vestibular dysfunction in children and adults; therefore, preoperative vestibular assessment (history-taking and physical examination) should be conducted before CI surgery, complemented with objective vestibular function tests, if necessary, to define the ear to be implanted and to be used for a comparative analysis of vestibular function postoperatively. (Strong Recommendation – Moderate-Quality Evidence).
XVII Due to an increased risk of falls in older people due to weaknesses in other systems, preoperative vestibular assessment for CI should be performed in these patients. (Strong Recommendation – Low-Quality Evidence).
XVIII Among the various tests, cHIT, vHIT, and rotary chair testing are the best tolerated by pediatric patients. (Weak Recommendation – Moderate-Quality Evidence).
XIX Children undergoing CI surgery have experienced vestibular deterioration postoperatively, especially in the cVEMP and caloric tests, suggesting that the otolith organs are more affected and that low-frequency tests are more sensitive than high-frequency tests for the analysis of the impact of CI on the vestibular system. (Strong Recommendation – Low-Quality Evidence).
XX Adults undergoing CI surgery have experienced vestibular deterioration postoperatively in the cVEMP and caloric tests, which can be used for this group. (Strong Recommendation – High-Quality Evidence).
XXI Adults undergoing CI surgery have not experienced vestibular deterioration postoperatively in vHIT, posturography, or DHI, with these tests providing lower diagnostic yield for this purpose. (Weak Recommendation – High-Quality Evidence).

### Early rehabilitation

Achieving age-appropriate spoken language in pediatric CI users has been associated with higher levels of nonverbal intelligence and maternal education, higher levels of residual hearing after surgery, early intervention, a focus on auditory and oral instruction, and the use of newer speech processor technologies.^94^ Improved educational attainment and quality of life have been described in children receiving a CI.^95^ However, among CI users, long-term educational and occupational levels continue to be significantly lower than the population average.^96^

The main goal of NHS programs is to diagnose hearing loss as early as possible and refer the infant to a specialized center to confirm the diagnosis, investigate the etiology, and initiate appropriate treatment. Every effort should be made to limit the period of auditory deprivation, encouraging treatment as early as possible. Hearing rehabilitation using PSAPs before 6 months of age significantly improves vocabulary, speech intelligibility, general language skills, and socioemotional development.^8^, ^97^

Children who receive a CI in infancy will benefit from auditory stimulation and demonstrate development in the brainstem and the thalamocortex.^98^, ^99^ However, if deprived of access to sound, the auditory cortex will experience developmental arrest and demonstrate cortical reorganization over time.^100^ Children with bilateral hearing loss receiving only unilateral CIs will also demonstrate reorganization in the form of aural preference toward the implanted ear. There is evidence supporting that a longer inter-implantation delay (sequentially) increases asymmetry in access to sound.^96^, ^101^

In children with congenital hearing loss, CI before 12-months of age offers the opportunity to promote auditory development during infancy and early childhood. The development of neural connections, a process known as functional synaptogenesis, peaks within the auditory cortex at around 2–4 years of age.^102^ Synaptogenesis decreases over time, a phenomenon that supports the idea of a critical period for the cochleotopic organization of the developing auditory cortex. The immature brain, with its increased plasticity, is especially suited to benefit from early CI.^103^ Therefore, access to sound and appropriate therapy during early childhood offers lifelong benefits to children with hearing loss.^104^

Lack of auditory stimulation can cause deviations in neural development, with long-lasting effects on auditory development, language acquisition, and even higher-level cognitive skills.^105^, ^106^ Likewise, unilateral auditory stimulation may cause asymmetric development and compromise how the auditory system responds to stimulation from a subsequent CI in the contralateral ear.^107^ Early CI improves auditory processing skills.^108^

CI in the first year of life has been associated with age-appropriate language gains, while comparatively poorer developmental outcomes have been observed in children undergoing surgery later in life.^109^ After 1-year of age, hearing gains decrease as the child’s age at the time of surgery increases. Colletti et al.^110^ found evidence of an additional benefit when CI is performed even earlier, before 6-months of age.

Although age at the time of CI may have only minor long-term effects on general language understanding, it has a lasting effect on more specific and complex skills within the domains of phonetics, expressive vocabulary, grammar, and semantics. These skills depend on the functional specialization of certain networks in the brain that are triggered by sensory input during early sensitive periods of neuronal development.^110^

While studies of central auditory development reflect the importance of a sensitive period for cortical plasticity that peaks around 2–4 years of age, the functional importance of CI in children under 12 months of age is highlighted by studies specifically investigating expressive and receptive language outcomes. Comprehensive literature reviews on language development in children with congenital hearing loss suggest that early CI may prevent long-term spoken language deficits in children implanted before 12 months of age.^111^, ^112^

Colletti et al.,^113^ investigating a cohort of 19 children implanted before 12 months of age, compared their speech and language development over 10 years of follow-up with that of children implanted after 12 months of age. The authors found that, after 5 years of CI use, all 19 children implanted before 12 months of age had achieved speech that was intelligible to the average listener, compared with only 67% of children implanted between 12 and 23 months of age.

Dettman et al.,^114^ evaluating a cohort of more than 400 pediatric CI users, found that children implanted before 12 months of age demonstrated significantly better speech perception, language acquisition, and speech production accuracy and were also more likely to score within the normative range of language performance upon school entry.

Bruijnzeel et al.^115^ conducted a systematic review of studies investigating potential speech and language benefits for children undergoing CI surgery before 12 months of age. The authors identified 14 studies and found that children implanted before 12 months of age performed better on speech production, auditory performance, and some receptive language scores. Ching et al.,^116^ in a prospective study of 350 children with hearing loss, found a strong positive benefit to early intervention; for example, children implanted at 6-months of age had significantly higher global language scores (a summary score of 20 language measures) at 5-years of age than those implanted at 24-months of age.

In infants, all tests should support a diagnosis of bilateral profound sensorineural hearing loss. Any inconsistencies in the testing should be addressed before making any recommendation. Evidence-based guidelines suggest that infants with a pure tone average better than 65 dB HL should continue with hearing aids, while infants with a pure tone average poorer than 80 dB HL should proceed to CI.^117^ Multidisciplinary CI teams should often closely monitor infants with hearing thresholds between 65 and 80 dB HL.^117^

Protocols for hearing aid fitting in infants with permanent hearing loss include monitoring of auditory and language development with parental reports/questionnaires, in addition to therapy to promote language.^118^ Teams should be cautious about proceeding to CI when infants are making progress in language development with consistent hearing aid use. Using a family-centered approach, multidisciplinary CI teams can help the child’s caregivers decide upon the best approach for language development, considering the unique characteristics of each family. Identifying an etiology of deafness, such as congenital CMV infection or genetic mutation, can help inform decision-making and reinforce confidence in the results of both objective and behavioral measures of hearing in infants.^119^

### Bilateral cochlear implantation in children

Binaural hearing is the ability of the auditory system to use sound information from both ears. In normal patients, it is considered to play an important role in speech recognition in noise, sound localization, and 3-dimensional perception of the sound environment. Three auditory mechanisms are responsible for the efficacy of binaural hearing: the head-shadow effect, the squelch effect, and the binaural summation effect.^120^

Bilateral implants are the most viable option for spoken language development.^121^ Simultaneous bilateral CI, compared with unilateral or sequential surgery, improves auditory performance, auditory pathway development in the brain, and, consequently, spoken language acquisition.^109^ If sequential CI is selected, the need to rapidly implant the second CI should be discussed with the family, which should occur within a few months of the first implant (up to 6-months),^122^ in addition to the use a PSAP on the non-implanted ear until surgery.^34^

Better language outcomes can be expected for children who receive early bilateral CIs.^123^ Therefore, it seems likely that the best intervention to promote oral language development for children with profound hearing loss is early simultaneous bilateral CI.^124^ Children with bilateral CIs achieve better language outcomes than those with unilateral CIs.^125^, ^126^ Young children spend most of their time learning and socializing in noisy environments.^127^ In such situations, bilateral stimulation helps reduce listening effort and facilitates learning.^125^, ^126^, ^128^

Patients using a unilateral CI without contralateral residual hearing have, by definition, a monaural hearing system. They have difficulty locating sound sources and understanding speech in noisy environments, a common situation in everyday life. These data support the increasing appeal in recent years for bilateral CI. Several authors have evaluated the performance of adult users of bilateral CIs on objective auditory tasks, most often through speech discrimination and spatial localization tests, in addition to self-assessment of auditory skills and quality of life.^120^, ^129^, ^130^, ^131^

Recognition between speech and noise when sound sources are spatially separated is more accurate in bilateral than in unilateral CI conditions because of the head-shadow effect. Better results in bilateral conditions are also observed for speech discrimination in quiet, which may be partly explained by the binaural summation effect and the increased likelihood of correctly detecting the signal. For spatial localization tasks, performance measured in bilateral conditions is significantly better than that measured in unilateral conditions.^132^ The patients who perform best in localization tests are also those who achieve the best scores in speech discrimination in noisy environments.^120^

Studies comparing the benefits of bimodal devices and bilateral CIs in oral language skills (receptive or expressive language) suggest that, in children with profound bilateral hearing loss, bilateral CI should be indicated. Children with thresholds close to 70 dB in one ear and profound hearing loss in the contralateral ear may benefit from bimodal stimulation. However, they should be closely monitored due to the risk of worsening hearing in the ear using the PSAP.^34^, ^133^

### Bimodal stimulation

A difficult issue for unilateral CI users may be the decision to pursue a second CI. Two implants provide the benefits of binaural hearing, including sound localization, binaural summation and squelch, and speech recognition in noise.^134^ CI users with residual hearing in the contralateral ear can use PSAPs (bimodal stimulation). However, for patients who are not clearly receiving bimodal benefit, the decision to pursue a second CI is not straightforward. Gifford and Dorman,^134^ comparing speech recognition between adults using bimodal stimulation and adults with bilateral CIs, observed no significant differences in speech perception or binaural summation using traditional clinical measures. The patient’s perception of the need for a second CI appeared to be of greater clinical utility than routine objective testing.

#### Recommendations

XXII NHS programs should diagnose hearing loss as early as possible and refer the infant to a specialized center to confirm the diagnosis, investigate the etiology, and initiate appropriate treatment. Ideally, the entire process should occur until 3-months of age to reduce the period of auditory deprivation. (Strong Recommendation – Moderate-Quality Evidence).
XXIII Adequate diagnosis, with TBABR or ASSR, assists in the use of PSAPs as early as possible at 3-months of age. (Strong Recommendation – Moderate-Quality Evidence).
XXIV Bilateral CI should be performed before 12-months of age in children with a diagnosis of bilateral profound sensorineural hearing loss. (Strong Recommendation – High-Quality Evidence).
XXV Infants with a pure tone average better than 65 dB HL should continue with hearing aids, while infants with a pure tone average poorer than 80 dB HL should proceed to CI. (Weak Recommendation – Moderate-Quality Evidence).
XXVI Multidisciplinary CI teams should often closely monitor infants with hearing thresholds between 65- and 80-dB HL. (Weak Recommendation – Moderate-Quality Evidence).
XXVII In children with bilateral sensorineural hearing loss, simultaneous bilateral CI should be performed if possible. (Strong Recommendation – Moderate-Quality Evidence).
XXVIII In children with bilateral sensorineural hearing loss, if sequential CI is selected, the second CI should be implanted within a few months of the first implant (up to 6-months), in addition to the use of a PSAP on the non-implanted ear until surgery. (Strong Recommendation – Moderate-Quality Evidence).
XXIX Bimodal stimulation can be used in children and adults, with monitoring of the hearing thresholds on the ear using a PSAP due to the risk of progressive hearing deterioration. (Strong Recommendation – Moderate-Quality Evidence).

### Cochlear implant indications

Indications for CI have increased substantially in recent years. Over the past few decades, electrode design and surgical techniques have evolved so much that hearing preservation is now possible. Current indications for adult CI are primarily based on open-set speech recognition. Typically, less than 50% of speech recognition scores are required for patients to meet CI candidacy. These score requirements are complex and vary slightly among centers in each country.

As the criteria for CI continue to broaden, the materials and methods used to test potential CI candidates remain highly variable and open to interpretation, even for traditional candidates with bilateral deafness. There are several parameters to help determine the suitability of a referral to CI and predict the likelihood that the applicant will qualify. The use of objective audiological criteria helps hearing care professionals make high-throughput and appropriate CI assessment referrals. However, it is essential to recognize that there is a poor correlation between unaided speech recognition scores and eventual CI application. Therefore, if a patient demonstrates limited benefit from appropriately fitted hearing aids, it is essential to consider referral for a formal CI assessment, regardless of performance on any single test or group of test measures.

The sentence recognition test has been the standard for measures of assisted speech in CI candidacy tests. Sentence recognition scores reflect less on how well a person can detect and process spectral and temporal components of speech and more on how well someone can use “top-down processing” to “fill in the missing pieces”. Cognitive features can also alter top-down processing. Post-deaf lingual voters score much higher on the sentence test than on the word test during the CI assessment.^15^, ^135^

When monosyllabic word recognition scores are used for CI candidacy qualification, performance results show significant improvement from pre- to post-CI. There is a trend towards better performance in the CNC word test when less restrictive criteria (<40% CNC) are used for application qualification rather than more restrictive criteria (<30% CNC).^15^, ^136^ Patients with bilateral deafness who undergo implantation in their worst ear, using a CNC score of ≤50% in the ear to be implanted, had a sensitivity of 99.7% to identify candidates who ultimately qualified based on previous CMS criteria (≤40% sentence recognition test). Pre- and postoperative CNC word score comparisons in the implanted ear demonstrated a significant improvement for those who scored up to 50% preoperatively.^137^

One of the best-assisted speech recognition tests is defined as the speech perception score in the individual ear(s) using optimized hearing aid(s) on a monosyllabic word test (Consonant-nucleus-consonant, CNC).^138^ The target presentation level for stimuli is 60 dB A.^139^ When unaided hearing thresholds are 60 dB HL or better in the untested ear, tamponade and muffling or masking using noise in the form of speech is performed. Patients who score ≤50% on the CNC in the ear with worse hearing should be considered for CI unless contraindicated for other reasons during CI evaluation. The CNC word score in the contralateral ear should not be considered when determining candidacy using this test.^137^

Communicating with background noise is a universal challenge because there is so much noise around us. Speech-in-noise tests have become integral to CI candidacy assessments and post-surgery monitoring. Cochlear implant candidacy guidelines are based on an individual's degree of hearing loss and assisted speech perception scores. These results are necessary to document that an individual is not receiving sufficient benefits from acoustic amplification alone and, therefore, may benefit more from a CI.

Early indications specified the use of HINT; however, clinicians often completed the evaluations in silence rather than performing the test in the adaptive SNR format as originally designed. Therefore, the test was not representative of hearing difficulties in real-world settings. Subsequently, the research identified that the HINT was prone to ceiling effects when the trial was completed only in silence to assess the pre-implant versus post-implant benefit. Updated recommendations based on the revised Minimum Speech Test Battery (MSTB 2011) suggest using the AzBio sentence test instead of the HINT.^140^ It has been recommended that tests be completed in both silence and noise to better document an individual's performance in a more realistic range of listening scenarios.^15^, ^16^, ^137^

The aided speech perception test usually includes CNC words, silent AzBio sentences, AzBio sentences in noise, and the BKB-SIN (AzBio sentences provide a correct percentage score at a fixed level, while BKB-SIN provides an SNR50 score). A presentation level of 60 dB SPL is recommended for all speaking material; however, when administering AzBio in noise, the clinic may choose its own SNR based on that clinic's candidacy evaluation protocol.^137^

CI candidacy assessments should be performed using appropriately fitted and verified HAs, i.e., using real ear or simulated real ear measurements conducted in a test box or at the ear to confirm that the devices meet the prescriptive goals for sufficient audibility. The Best Aided Connected Speech Test (best aid defined as speech recognition test in the individual ear using optimized hearing aid) should be performed using AzBio phrases to determine if a patient qualifies for coverage for their CI. There is no consensus on the recommended SNR for testing, and it varies between clinics. Zitler et al.^137^ recommend testing the ear to be implanted in the best aid condition using AzBio phrases in noise starting with an SNR of +10 dB using a babbling of 10 speakers (AzBio phrases presented at 65 dB A and noise presented at 55 dB A). To better assess auditory status, the clinician should consider decreasing the adversity (phrases presented silently at 60 dB A) or increasing the adversity (phrases at +5 dB SNR with phrases presented at 65 dB A and noise presented at 60 dB A) of the hearing condition, as needed.

Although clinically meaningful improvements in speech perception in noise can be achieved after surgery for CI-eligible patients in noise, improvements tend to be smaller as the SNR becomes more adverse.^141^ People qualified for CI only in the SNR condition of +5 dB can obtain significant benefits from their device, but the objective results are more variable.^142^ Consideration of hearing needs and goals should be discussed with each candidate individually. The variation in SNR represents real-life hearing situations and may be beneficial in determining SNR in which substantial difficulty is noted. Testing AzBio Sentences on the person's daily hearing condition is recommended for postoperative comparison with the person's daily hearing condition. The daily hearing condition is defined as a test with the optimized auditory configuration typical of a patient's daily hearing (e.g., unilateral or bilateral non-occluded hearing aid(s), bimodal, unilateral or bilateral CI(s), EAS with contralateral HA).^137^

The decision on which ear to implant is differentiated, and it is not possible to create a single approach that leads to specific recommendations. Although the ear with the worst hearing has been routinely selected in the past, dogmatic methodology should not take precedence in all cases over these other factors.^143^ This less rigid approach may lead to a recommendation for implantation of the ear with better hearing in some cases when it also meets the candidacy. When the examination indicates significant pathology in one ear, implantation of the uninvolved ear (which may be the ear with the best hearing) is often indicated. However, in cases with a non-revealing history, normal examination and imaging, and symmetrical auditory thresholds, the ear with the worst performance on the CI candidacy test is routinely selected for CI.

Although the list of indications is expanding, contraindications to CI are becoming clearer as time passes and studies emerge. First, patients need a cochlear nerve to undergo CI. MRI is used to determine the presence of a cochlear nerve in children with congenital deafness; in adults, it can diagnose tumors or demyelination. Although definitive absence of a nerve is not always easy to determine, imaging that indicates absence or hypoplasia of a cochlear nerve is a relative contraindication to CI. The duration of deafness is another important factor. Adult patients with prelingual hearing loss who have not developed language are considered a relative contraindication.

Some challenging decision-making is necessary over time as more adult patients with natural low-frequency hearing find CI a possibility for rehabilitation. Despite evidence of the potential to preserve low-frequency hearing, CI surgery also poses the risk of losing residual hearing, and the durability of hearing preservation remains unclear.^144^ In children, this decision is often not as relevant, although the decision to perform bilateral CI in single or sequential procedures has become relevant in this population. As the criteria for CI evolve, the decision-making process becomes more complex, requiring comprehensive discussions among patients, families, physicians, and speech therapists.

### Unilateral deafness

CI has been increasingly performed in patients with unilateral deafness or Single-Sided Deafness (SSD). Traditional treatments included the use of PSAPs with Contralateral Routing of Signal (CROS) systems and bone-anchored hearing aids. CI was initially discouraged due to the misconception that electric stimulation would not integrate with contralateral acoustic input. However, van de Heyning et al.^145^ demonstrated feasibility after implanting 21 patients with SSD and debilitating tinnitus. Indications for CI in patients with SSD have been expanded to include even children with congenital pathologies who have a normal cochlear nerve.^146^, ^147^ Adults have experienced tinnitus improvement and have become daily CI users.^148^ Overall, implanted patients with SSD experience a reduction in tinnitus in the implanted ear, in addition to improvements in speech perception in noise, sound localization, and quality of life.^146^, ^149^, ^150^

### Electric-acoustic stimulation

Previously, patients needed to have profound or total bilateral sensorineural hearing loss to be considered CI candidates. Technological advances have allowed the expansion of indication criteria to include individuals with significant levels of residual hearing. Because the goal of expanding CI indications is to maintain or preserve hearing, some patients with normal hearing or moderate low-frequency hearing loss and severe-to-profound high-frequency sensorineural hearing loss have used Electric-Acoustic Stimulation (EAS) or hybrid stimulation.^151^

Patients with residual low-frequency hearing often have poor outcomes with PSAPs. For these patients, CIs with EAS or hybrid CIs allow for low-frequency acoustic amplification and middle-to-high-frequency electrical stimulation.^152^ EAS improves speech recognition in noise and provides finer spectral resolution of complex auditory stimuli (e.g., music).^17^, ^151^

Low-frequency acoustic cues are important for speech perception and other auditory skills that are transmitted more effectively than electric information provided there is useful residual hearing. Hybrid CI users show significantly increased perception of sound frequency compared with electric-only stimulation.^153^, ^154^ Acoustic perception of low-frequency sounds allows patients to use the fundamental frequency and first formant of speech, timing cues, and spectral information more effectively than auditory electrical stimulation.^155^ The additional acoustic information can compensate for the limitations associated with the auditory stimulus generated by the CI, such as the reduction in the number of functional channels and poor pitch perception, leading to difficulty when listening in competing voice situations, even for CI users who achieve high levels of speech recognition in quiet.^156^

It is essential to use an atraumatic surgical technique, including a very delicate opening of the cochlea, with slow insertion of thin electrodes that are shorter than usual.^157^ This indication is particularly important for older patients, as aging also reduces hearing clarity with a well-fitted hearing aid.^158^ In these patients, postoperative EAS (low-frequency acoustic stimulation and high-frequency electrical stimulation) allows for a better understanding of speech in noise and the perception of music. CIs can improve speech understanding for this type of patient. Electrode length has been identified as a critical factor: greater insertion depth improves speech perception in “traditional” candidates but may destroy residual hearing. Shorter electrodes, however, may compromise outcomes for patients who ultimately lose residual hearing and require electric-only stimulation.^159^, ^160^

Optimizing insertion depth (∼360 °), thinner electrodes, and Round Window (RW) insertion (vs cochleostomy) are strategies for “soft” surgery.^156^, ^161^, ^162^ Intraoperative monitoring using Electrocochleography (ECochG) has also become available as an adjunctive tool to minimize intracochlear trauma.^163^, ^164^, ^165^ Intraoperative ECochG may be obtained directly through the CI during insertion. Acoustic stimulation is provided by inserting an earphone, whereas cochlear hair and neural cell responses are detected through the inserted electrodes.^165^ The recorded signal provides intraoperative feedback on cochlear hair cell function and may influence the insertion technique or insertion depth when hearing preservation is intended.

Pillsbury et al.,^166^ in a prospective multicenter study of 73 patients with residual low-frequency hearing and severe-to-profound hearing loss in the middle-to-high frequencies treated with EAS, reported that 85% of patients performed better on the Speech Perception Score (SPS%) at 12-months with EAS use compared with the preoperative aided condition. Achena et al.^158^ reported that the mean percentage of SPS in quiet changed from 21.8% before surgery to 71.8% after surgery, while in noise it changed from 17% to 65% (*p* < 0.001). The indication criteria for hybrid CI have included the following parameters:^144^, ^167^

a) Audiometric thresholds less than 65 dB at frequencies from 125 to 500 Hz and greater than 75 dB at frequencies above 1000 Hz;
b) Sensorineural hearing loss with air-bone gap less than 15 dB, if present;
c) Result of the speech perception test, in quiet, less than 50% in the better ear, with the best possible amplification conditions;
d) Stable hearing loss for more than 2-years;
e) Having received counseling about CI expectations.

#### Recommendations

XXX Speech-in-noise tests are essentials to CI candidacy assessments and post-surgery monitoring, but there is no consensus on the recommended SNR for testing. Strong Recommendation – High-Quality Evidence.
XXXI The Best Aided Connected Speech Test (defined as speech recognition test in the individual ear using optimized hearing aid) could be performed using AzBio phrases to determine if a patient qualifies for coverage for their CI. Strong Recommendation – Low-Quality Evidence.
XXXII The indication of CI in children with congenital hearing loss and adults should be preceded by the use of a PSAP, but in children with a genetic diagnosis of profound deafness and patients with hearing loss caused by bacterial meningitis, the use of a conventional hearing aid is not necessary. (Strong Recommendation – High-Quality Evidence).
XXXIII Adults and children over 12-years of age should have an established language code before being referred for CI surgery. (Strong Recommendation – Moderate-Quality Evidence).
XXXIV CI can be used in children and adults for the rehabilitation of profound unilateral sensorineural hearing loss, especially in the presence of refractory disabling tinnitus. (Strong Recommendation – Moderate-Quality Evidence)
XXXV When performing CI surgery for electric-acoustic stimulation, it is recommended to use strategies in the surgical technique to preserve the patient's residual hearing. (Strong Recommendation – High-Quality Evidence).

## Discussion

### Surgery

CI surgery is safe if performed by experienced surgeons. Although a variety of techniques have been described to access the cochlea, the most commonly used approach is posterior tympanotomy.^168^ It allows access to the RW membrane and cochlear promontory with a direct route for electrode insertion with few serious complications.^169^ Device fixation techniques have become less frequent as the subperiosteal pocket has become more popular.^170^ The use of the subperiosteal pocket decreased the likelihood of rare but potentially devastating intracranial complications reported with cortical drilling for fixation.^171^, ^172^

Facial nerve monitoring is recommended in almost all otologic and neurotologic procedures that involve the intrapetrous facial nerve,^161^, ^162^, ^163^, ^164^, ^165^, ^166^, ^167^, ^168^, ^169^, ^170^, ^171^, ^172^, ^173^ and, although damage to the patient or the implant itself has not been demonstrated, there is concern and risk of inducing electric current through the CI electrode with monopolar cautery. Because of this risk, monopolar cautery should not be used routinely in patients with an existing CI.

Other techniques have been described, but they are largely reserved for patients with anatomic variations that require deviation from the facial recess.^174^ The most frequently described alternative approach is the suprameatal approach,^175^, ^176^, ^177^ which requires a tympanomeatal flap to be raised to access the RW. This approach avoids the need to drill the facial recess and thus reduces the risk of injury to the facial nerve.^178^ The combined approach technique described by Lavinsky et al. can also be used,^160^ in which a minimal, reduced posterior tympanotomy is performed, only large enough to pass the electrode cable, with the RW approach or cochleostomy being performed via the transcanal route. Therefore, in special cases such as malformations, it allows a safe approach via posterior tympanotomy to pass the cable providing a wide transcanal surgical field for insertion of the electrode array. Rarely used techniques such as middle fossa and closure of the External Auditory Canal (EAC) have been described for CI, but they are not routinely used.^161^, ^162^, ^163^, ^164^, ^165^, ^166^, ^167^

Regardless of the surgical approach, the goal is to place the CI electrode within the Scala Tympani (ST). Placement within the ST optimizes the interaction between the electrode and the spiral ganglion cells, which are being directly stimulated. It has been well demonstrated that user outcomes are improved with electrodes in the ST, 179, 180 and any translocation to the scala vestibuli decreases CI performance. In addition, as soft surgery techniques become standard, RW membrane insertions have been favored because they avoid the potential trauma of drilling a cochleostomy.^162^

#### Recommendations

XXXVI Although there are other techniques to access the RW, an approach via posterior tympanotomy is the most recommended for CI surgery. (Strong Recommendation – Low-Quality Evidence).
XXXVII Electrodes should be placed within the ST of the cochlea. Therefore, insertion through the RW is preferable if possible. (Strong Recommendation – Low-Quality Evidence).
XXXVIII Using a subperiosteal pocket may be an option to accommodate the internal component. (Moderate Recommendation – Moderate-Quality Evidence).
XXXIX Monopolar electrocautery should not be used after the internal device enters the surgical field or when performing CI surgery on a second side. (Moderate Recommendation – Low-Quality Evidence).
XL Intraoperative facial nerve monitoring (IFNM) is routinely recommended and is essential in patients with inner ear malformations due to a major risk of affecting the anatomy of this nerve. (Strong Recommendation – Low-Quality Evidence).

### Cochlear implant in infants under 1 year of age

Additional intraoperative care is required for patients younger than 12-months. Pediatric patients have significantly lower blood volume than adult patients. Children younger than 12 months have a total systemic blood volume of approximately 80 mL/kg. Minor blood losses in the pediatric population can have catastrophic effects. Blood losses greater than 10% in this population can lead to hypovolemia.^108^, ^181^ Bleeding from emissary veins and bone marrow oozing from the temporal bone are the main sources of blood loss during pediatric CI.^182^ Care should be taken to maintain meticulous hemostasis throughout CI surgery in children younger than 12-months to avoid potential complications.

The skin of children younger than 12-months is significantly thinner than the skin of adults. Flaps should be manipulated with caution.^108^, ^183^ Skull thickness of younger pediatric patients may be less than a few millimeters. Dura mater exposure during pocket creation in the temporal bone cortex can be performed to reduce tension under the flap and improve aesthetic outcomes.^170^ Receptors have become increasingly thinner, making it possible to perform it from the pocket. Because the skull develops over time and migration of the internal component may occur, the device should be well positioned and the pocket should be created properly.^31^, ^170^, ^182^

#### Recommendations

XLI CI in infants under 1-year of age is safe and effective. The rates and nature of major and minor complications are comparable to those in studies of adults and older children. (Strong Recommendation – Moderate-Quality Evidence).

### Hearing preservation

Lehnhardt first described the concept of “soft surgery” cochleostomy (minimal cochleostomy inferior and anterior to the RW) in 1993.^184^ The principles of the hearing preservation technique include no perilymph suctioning, careful opening of the cochlea, slow and gentle insertion of electrodes, and use of intraoperative corticosteroids to reduce foreign body reaction. Hearing preservation has been reported in up to 90% of patients.^156^, ^185^, ^186^ Antibiotic prophylaxis may prevent biofilm formation on the electrode surface.^185^

Postoperative loss of residual hearing is linked to the direct physical trauma caused by electrode insertion and the associated acute inflammatory response. Postoperative hearing loss may be linked to chronic inflammation and the development of fibrotic tissue in the cochlea (in the long term).

The atraumatic electrode insertion is an important factor for hearing preservation. The following factors should be considered^187^:

1) Electrode insertion depth.
2) Electrode array insertion forces against intracochlear structures.
3) Selection of electrodes best suited to the individual patient’s cochlear anatomy, especially in the presence of inner ear malformations.

Electrode insertion via the RW requires a shorter drilling time in the cochlea and may result in less acoustic trauma. A systematic review of 16 studies comparing the RW approach vs. cochleostomy concluded that both insertion approaches resulted in comparable hearing preservation.^156^ However, some studies using window insertion showed better hearing preservation outcomes (about 20% higher)^188^ than those in which cochleostomy was used.^156^

ECochG can be used to monitor electrode array insertion in real time. Reduced ECochG responses after implantation are expected as a result of intracochlear trauma and mechanical changes induced by electrode insertion.^189^ However, ECochG amplitude responses after insertion may increase.^163^ There is a high degree of correlation between ECochG responses and speech perception performance in adults.^190^

The speed of electrode insertion is important. A slow and steady insertion speed of approximately 30 seconds reduced forces within the cochlea compared with faster insertion speeds.^191^ Insertion speed appears to have a significant impact on inner ear fluid dynamics and hearing and vestibular preservation. The optimal insertion speed remains unclear.^192^ Hyaluronic acid has been used as a lubricant to reduce trauma during electrode array insertion, but it has no benefit for hearing preservation.^193^

Although shorter electrodes may have better rates of early hearing preservation,^156^ complete electrical stimulation of the cochlea may not be possible in cases of residual hearing loss. Friedmann et al.^194^ reported significantly better speech understanding after loss of residual hearing in patients implanted with the CI422 (Cochlear) than in patients implanted with the shorter electrode. Hearing preservation with full insertion of a standard-length electrode is also possible.^193^ The advantage of using standard electrodes is the possibility of stimulating the distal cochlea if hearing loss progresses after surgery.

The use of steroids is common in protocols to reduce hearing loss caused by inflammation.^156^, ^188^, ^193^, ^195^ The optimal timing of administration of corticosteroids also remains to be established. The use of preoperative steroids combined with intraoperative steroids has a positive impact on hearing preservation compared with other regimens or no steroid use at all.^188^ Topical steroid application has been associated with better hearing preservation outcomes, presumably by reducing the electrode-induced acute phase response.^192^

Parameters to define hearing preservation^156^:

    I Complete hearing preservation: postoperative loss in hearing thresholds of a maximum of 10 dB at each frequency to be preserved;
    II Partial hearing preservation: postoperative loss in hearing thresholds above 10 dB at each frequency, but with residual hearing less than or equal to 80 dB for at least one frequency between 250 and 1000 Hz;
    III No hearing preservation: the patient is unable to use acoustic stimulation because of thresholds worse than 80 dB.

### Recommendations

XLII Hearing preservation in CI surgery should be encouraged for better device performance, reducing the risk of vestibular trauma. (Strong Recommendation – Moderate-Quality Evidence).
XLIII Slow and gentle insertion of electrodes through the RW and no perilymph suctioning are the principles of hearing preservation surgery. (Strong Recommendation – Low-Quality Evidence).
XLIV The use of topical corticosteroids in the RW is associated with better hearing preservation. (Moderate Recommendation – Low-Quality Evidence).

### Electrodes

All major CI manufacturers have their own electrode design that addresses patient and surgeon needs. The 3 main factors for the quality of insertion of the CI electrode array are insertion depth, proximity to the modiolus, and placement within the ST.^196^ The 2 types of commercially available CI electrode arrays are lateral wall and precurved (perimodiolar). The electrode known as “Mid-Scala” is considered perimodiolar.

#### Perimodiolar electrodes

Perimodiolar electrodes are associated with an increased risk of trauma during insertion due to damage to the spiral ganglion cells, in addition to increased likelihood of translocation to the scala vestibuli with the removal of the stylet that holds the electrode straight before insertion into the cochlea.^180^, ^197^ Many surgeons have refined their techniques to make the insertion procedure as atraumatic as possible by adjusting the speed of insertion and carefully pulling the stylet out of the cochlea. However, such techniques are not always consistent across different surgeons.^198^ Trauma to the basilar membrane can result in osteogenesis and fibrosis, which can reduce hearing gain.

Perimodiolar electrodes have a shallower insertion depth because they cannot be made longer than 16–18 mm and cannot be inserted deeper than 380°–420 °. For an average sized cochlea, 420 ° of insertion depth would cover a frequency range of up to 500 Hz. One argument for using precurved electrodes is that they can reach the spiral ganglion cells. However, these cells extend up to 660 ° of insertion depth,^199^, ^200^ which is not reached by any perimodiolar electrode currently available on the market. In addition, perimodiolar electrodes have predefined curvature dimensions that may not fit into every cochlea, and as a result, the perimodiolar electrode may not be a true perimodiolar electrode in every cochlea.^201^

#### Lateral wall electrodes

Lateral wall electrode arrays are available in a wide range of lengths. Because no stylet is required for insertion, the risk of trauma is significantly reduced compared with perimodiolar electrodes.^180^, ^197^, ^202^ However, in some cases, achieving full insertion may still be challenging for the surgeon.

There is no consensus in the literature on whether there is a relationship of closer proximity of the electrode contacts to the modiolus with auditory performance^203^ and battery life.^204^ Electrode array extrusion is a concern with straight electrodes; therefore, the surgeon must be extremely careful when inserting the electrodes.

Insertion depth of an electrode array within the cochlea depends on the length of the patient’s cochlear duct and the selected electrodes. Full insertion into the cochlea is not always possible for a number of reasons, such as the angle at which RW opening was performed, anatomic variations of the cochlea, and obliteration of the cochlear duct. The surgeon’s goal should be to place all electrodes inside the cochlea without causing any damage. There is some disagreement about the relationship between insertion depth and user performance, although a greater correlation is observed between deep insertion and better hearing performance.^180^, ^205^, ^206^

Other factors that influence the hearing performance of CI users include the absence of spiral ganglion cells in certain regions of the cochlea known as “dead regions”, long-term deafness, and malformed cochlear anatomy.^207^ Partial coverage of electrical stimulation in CI recipients with profound postlingual deafness may lead to a frequency mismatch between the position of the intracochlear electrode and the location of the characteristic frequency of the neural elements.^208^

A flexible electrode that applies less force to the intracochlear blood vessels may increase the likelihood of long-term hearing preservation,^209^ compared with a stiffer electrode that may result in the application of more force to the surrounding blood vessels, thereby limiting the blood supply to the neural elements that extend into the apical portion of the cochlea where residual hearing is typically located.^210^

An electrode array that is larger in dimension would take up more volume at the apex of the cochlea and could also produce higher intracochlear pressure depending on the speed of electrode insertion into the cochlea.^211^ However, a larger electrode array would also bring the stimulating contacts closer to the neural elements, with the opposite effect occurring with a thinner electrode. Therefore, a balance is needed to determine how far the stimulating contacts should be positioned from the neural elements.

The normal-functioning human cochlea can hear sound signals in the frequency range of 20 kHz in the basal region to 20 Hz in the apical region. There is debate as to whether the electrodes stimulate the neural endings in the organ of Corti or the spiral ganglion cell bodies directly. Lateral wall electrodes are believed to stimulate the nerve fiber endings in the organ of Corti where the electrode array is located, whereas perimodiolar electrodes stimulate the spiral ganglion cells. Therefore, insertion depth may be important. The organ of Corti extends throughout the cochlea, whereas the spiral ganglion cells within Rosenthal’s canal extend 1.75–1.85 turns of the cochlea.^187^

#### Recommendations

XLV Perimodiolar electrodes are less likely to extrude than lateral wall electrodes. (Strong Recommendation – Low-Quality Evidence).
XLVI Patients with cochlear malformations, such as CC deformity, should not use perimodiolar electrodes. (Strong Recommendation – Moderate-Quality Evidence).
XLVII Because lateral wall electrodes are relatively larger, they achieve greater insertion depth than perimodiolar electrodes. (Moderate Recommendation – Low-Quality Evidence).

### Telemetry

There are several methods to attempt to obtain objective measurements of auditory nerve function after electrical stimulation in CI users. The most commonly used procedure during surgery is the electrically Evoked Compound Action Potential (ECAP). ECAP thresholds can be useful to predict the minimum and maximum levels that should be used in mapping the electrodes for programming the speech processor, facilitating this process in children, and determining the stimulation parameters that will result in programming with more appropriate levels, which can improve user performance.

The ECAP is recorded on the CI using specific software, called telemetry system. Telemetry is a mechanism for capturing distant events that consists of 2 systems. The first is used to measure the impedances of each electrode, monitoring the suitability of electric current generators. The second system, Neural Response Telemetry (NRT), allows the recording of the ECAP of the distal portion of the auditory nerve in CI users, using the implant itself to elicit the stimulus and record the responses.

Impedance is related to the resistive characteristics of the fluid and tissue surrounding the electrodes and is one of the factors that determine the energy consumption of the CI system. Impedance telemetry should always be performed before NRT to confirm the proper functioning of the receptor and stimulator and to check for the occurrence of an open-circuit or short-circuit fault in the intracochlear electrodes by measuring their electrical resistance.

NRT is performed after surgery. The CI stimulates the cochlea, and the ECAP is recorded by amplifying the signals from the intracochlear electrodes. After checking the patient’s ECAP, a plain transorbital X-Ray is undertaken to confirm the position of the electrodes in the cochlea or CT, which is recommended in some services around the world. The patient returns for postoperative care common to other ear surgeries. After 4–6 weeks, the patient returns for adjustment of the external component of the implant and its programming or “mapping”.

#### Recommendation

XLVIII NRT should be performed after insertion of the electrode array to assess the integrity of the implanted device and patient responses. Strong Recommendation ‒ Low-Quality Evidence.

### Early activation of cochlear implants

When CI started, it was recommended to delay initial activation until at least four weeks after CI surgery due to possible complications and to avoid wound disruption.^212^ However, advances in surgical techniques and fitting experiences have allowed initial activation within 24 h. Emerging evidence suggests that early activation is feasible and beneficial, giving patients faster access to sound and rehabilitation.^213^, ^214^

Activation is considered early variably between studies (up to 14 days postoperatively). Some studies report activation within one day postoperatively.^215^, ^216^ Some studies have indicated low impedance levels in the early activation group.^216^, ^217^ Magnet force adjustment or using the processor outside the ear was often recommended. Complication rates are low, with no difference from late activation. Early activation has improved patient satisfaction and anxiety levels without impairing speech recognition or rehabilitation.^213^, ^215^, ^216^ Regular inspection of magnet strength is recommended as part of CI follow-up, as postoperative wound edema should be expected.^218^

Patro et al.^219^ evaluated 604 adult CI recipients, stratified by time of CI activation (Group 1: ≤10d, n = 47; Group 2: >10d, n = 557). CI activation within ten days of surgery is associated with increased early device use and superior speech recognition at early (AzBio in quiet performance at three months) and late (AzBio in quiet performance at 12-months) follow-up visits. The timing of activation and device use are modifiable factors that can help optimize postoperative outcomes in the CI population.

Alahmadi et al.^217^ compared the values of electrode impedance at different times within two years in patients who activated the CI early (one day after surgery ‒ n = 481) with patients who activated it four weeks later (classic activation ‒ n = 434). Early activation led to sustained reductions in electrode impedance compared to classical activation, suggesting that early activation may positively affect long-term CI outcomes.

#### Recommendation

XLIX Early activation of cochlear implants is feasiblewith low complication rates. Moderate Recommendation- High-Quality Evidence

### Vaccination and prophylactic antibiotic therapy

The estimated risk of (immediate and long-term) postoperative meningitis after CI is low, approximately 0.07%.^220^ However, it is higher than the risk of meningitis in the general population – 20 cases per 100,000 population (0.02%),^221^ but in countries such as the United States and the Netherlands, the rate is very low (<0.002%).^222^, ^223^ Bacterial meningitis is a disease with high morbidity and mortality rates. The most commonly detected organisms are *Streptococcus pneumoniae*, *Haemophilus influenzae*, and *Neisseria meningitis*. Even with adequate treatment, 5%–10% of patients die, and complications, such as severe brain injury, occur in 10%–20% of survivors.^224^ The pneumococcal meningitis mortality rate is even higher, ranging from 15% to 60%.^225^ Infants under 1-year of age would be at greater risk of having meningitis because of the immaturity of their immune system. However, systematic reviews and meta-analyses have not found such an association in this age group, with a negligible rate of meningitis.^226^

Some authors have suggested that Acute Otitis Media (AOM) may be a risk factor for post-implant meningitis.^221^, ^227^ Bacteria from the infected middle ear could cause meningitis by passing into the inner ear through the electrode array. Other studies suggest that the risk of meningitis in those who develop postoperative AOM remains negligible.^220^ However, in cases of AOM where CI surgery occurred within 2-months, there is a concern about an increased risk of meningitis due to the spread of the middle ear bacteria into the CSF via the cochlear perilymph.^228^, ^229^

The 2-main methods of electrode insertion are the RW approach and cochleostomy. A variant of the RW approach involves extending the edges of the RW prior to electrode insertion (extended RW technique).^228^ Recent evidence suggests no difference in cochlear trauma risk between these techniques, with improved speech perception using the RW approach.^212^ An international survey suggests that most surgeons now use the RW insertion approach.^230^ In a systematic review, the pooled rate of meningitis was negligible for both RW (1-case in 1557 patients) and cochleostomy (0 cases in 584 patients).^220^

The main agent involved in cases of CI-related meningitis is pneumococcus.^231^ There are more than 90 serotypes of *S. pneumoniae*. The maximum number of serotypes covered by vaccination is 23. Currently, there are 2 commonly available vaccines for *S. pneumoniae*: 1) Pneumococcal 13‐Valent Conjugate Vaccine (PCV13) (Prevnar 13, Pfizer Inc., New York, NY) and 2) Pneumococcal Polysaccharide Vaccine (PPSV23) (Pneumovax 23, Merck & Co., Kenilworth, NJ). A second dose of PPSV23 should be administered 5-years after the first.

The risk of meningitis is influenced by the combination of risk factors a patient has. Several risk factors can contribute to meningitis in CI users – otitis media, age, head trauma, surgical technique, cochlear malformations, and CSF leak.^220^ The CI electrode array is a route for spreading infection from the middle ear to the cochlea.

Given the underlying evidence of efficacy, pneumococcal vaccines are part of routine childhood immunization programs in many countries. It is recommended that CI candidates and users be fully vaccinated against the agents causing bacterial meningitis, especially *S. pneumoniae* due to its high prevalence. The U.S. Centers for Disease Control and Prevention (CDC) recommends that children with CIs should receive one dose of PCV15, followed by one dose of PPSV23.^232^, ^233^ In 2022, the CDC updated its guidelines to include that unvaccinated adults with CIs should receive one dose of a conjugate vaccine (PCV20). If PCV15 is used in this group, it should be followed by one dose of PPSV23.^234^ The immunization schedule varies depending on the country.

#### Prophylactic antibiotic therapy

Antibiotic prophylaxis is recommended after CI surgery.^235^, ^236^ Some systematic reviews and meta-analyses, such as Cochrane reviews, have highlighted that there is limited evidence that prophylactic antibiotics reduce postoperative infections in otologic surgery.^220^, ^237^, ^238^ While there is a lack of randomized clinical trials supporting this practice, this recommendation remains in place to prevent the potentially serious and expensive sequelae of infectious complications, including death and reimplantation.

A retrospective cohort study of 1180 patients found that those who received 48 h or less of antibiotics (antibiotic prophylaxis) were almost 3 times as likely to develop infection as patients who received more than 48 h of antibiotics (prolonged antibiotic therapy). Children had almost 4 times the risk of developing infection as adults, and the infection rate among children who received antibiotic prophylaxis (3.2%) was almost 3 times higher than that observed among children who received prolonged antibiotic therapy (1.2%), but no cases of meningitis were observed.^236^

#### Recommendations

L Patients who will receive a CI should be vaccinated against pneumococcus before surgery. (Strong Recommendation – Moderate-Quality Evidence).
LI Postoperative antibiotic prophylaxis is recommended for patients undergoing CI surgery. (Strong Recommendation –Low-Quality Evidence).
LII CI users with AOM should receive age- and weight-appropriate antibiotic therapy. (Moderate Recommendation – Low-Quality Evidence).

### Auditory neuropathy spectrum disorder

Hearing in mammals relies on the ability of sensory hair cells to convert sound-evoked mechanical stimuli into electrochemical signals. Hair-bundle deflection induces rapid opening of sensory transduction channels, leading to the generation of an influx of cations into the IHC. This results in a depolarizing potential, allowing an influx of calcium through voltage-gated calcium channels. The coupling of Ca2+ channels at the presynaptic site triggers synaptic vesicle fusion and neurotransmitter glutamate release into the synaptic cleft.^239^ The release of glutamate in the synapse activates Ca2+-sensitive AMPA receptors. This initiates the generation of stimuli in the spiral ganglion neuron fibers, which encodes information about sound stimuli that is sent to the central nervous system.^83^, ^240^

The synapse of the peripheral auditory system includes the presynaptic site (the base of the hair cell), the synapse itself, and the postsynaptic site (terminal dendrite of the spiral ganglion). The base of IHCs interfaces with terminal processes of spiral ganglion neurons. Presynaptic release of glutamate leads to the transmission of a sound signal to the spiral ganglion neurons and propagation of the signal in the central auditory pathway. The synapse between the IHC and the spiral ganglion terminal process consists of several unique features. First, each spiral ganglion neuron receives input from a single IHC, but each IHC synapses with multiple spiral ganglion neurons.

This synapse is able to encode a high degree of temporal precision due to graded release of glutamatergic vesicles via the synapse. The synapse consists of a long tether that holds synaptic vesicles close to the presynaptic release point in IHCs as it is anchored to the cell membrane at the presynaptic active zone in close proximity for release into the synaptic cleft. This presynaptic organization allows for transmission of a finely temporal encoded (up to 1 kHz) and indefatigable signal. The synaptic component also includes the postsynaptic glutamate receptors at the terminal dendrite of the spiral ganglion.

The neural partition of the human auditory system consists of the distal neurite of the spiral ganglion, the somata of the spiral ganglion that reside within Rosenthal’s canal, and the central processes of the spiral ganglion that form the cochlear partition of the vestibulocochlear nerve (eight cranial nerve) and branch to form synapses within the dorsal and ventral cochlear nucleus of the midbrain. Lossless conduction of this signal occurs through the myelinated distal and central dendrites of the spiral ganglion. Central components of the human auditory system include the brainstem and auditory cortex of the temporal lobe, which are together responsible for maintaining the tonotopic organization of sound and processing sound and speech, respectively.

A dysfunction at any level of this complex transduction machinery can make sound coding difficult. Potential sites of damage are diverse, including IHCs and their synapses with spiral ganglion neurons or synaptopathy (e.g., presynaptic glutamate release or postsynaptic terminal dendrites of spiral ganglion neurons), or can be due to demyelination and axonal loss of auditory nerve fibers and their targets in the cochlear nucleus (neuropathy). These auditory pathologies are called Auditory Neuropathy Spectrum Disorder (ANSD), in which the activity of OHCs is maintained.^240^, ^241^

It has long been hypothesized that individuals with auditory neuropathy would be poor CI candidates because transmission of the signal from electrical stimulation of the spiral ganglion through the CI could be affected. A study of 260 individuals with ANSD showed positive, albeit variable, CI outcomes in these patients.^83^, ^242^ Some individuals will have qualitative or quantitative deficits to the spiral ganglion or cochlear nerve and are hypothesized to have poor outcomes with CIs. However, some individuals with ANSD have a genetic lesion affecting the synapse itself, which would be bypassed by the CI, and these individuals would therefore be expected to have the same outcomes as those with a genetic lesion affecting IHCs.^243^, ^244^ Hearing loss is characterized by the presence of OAEs and/or Cochlear Microphonics (CM), indicating normal cochlear function, and by altered auditory responses from the brainstem measured by ABR indicating abnormal transmission of the synaptic signal to the central nervous system.^244^, ^245^, ^246^ Furthermore, absence of the stapedial reflex and contralateral suppression of OAEs are commonly observed.^222^, ^245^, ^247^

The CI bypasses the sensory and synaptic partitions by directly stimulating the spiral ganglion somata, leading to the transmission of electrical signals to the midbrain. Therefore, it is concluded that the health of the organ of Corti or synapse will not affect the outcomes of CI. Conversely, the health of the spiral ganglion (or more generally the auditory nerve), the synapse between the spiral ganglion and the cochlear nucleus, midbrain, or auditory cortex may negatively affect the electrical transmission of sound by a CI and, theoretically, could lead to suboptimal CI outcomes.^248^

Environmental causes of ANSD primarily affect newborns and include hyperbilirubinemia, thiamine deficiency, and hypoxia. ANSD can also be noise-induced or age-related. Spiral ganglion neurons are bipolar, with cell bodies located in the modiolus of the cochlea, peripheral axons running through the spiral limbus toward the row of cochlear IHCs, and proximal axons that synapse at the midbrain. These neurons are tonotopically organized with the cochlea.^242^

Multiple spiral ganglion neuron fibers synapse with each IHC. Their axons are myelinated and project to the brainstem. In response to glutamatergic stimulation at the synapse, excitatory postsynaptic potentials at the peripheral axon lead to gradual Na + influx via a large number of channels, which allows for temporal encoding of acoustic stimuli.^242^, ^248^

#### Nonsyndromic auditory synaptopathies

Syndromic auditory neuropathies affect multiple cranial and peripheral nerves, whereas nonsyndromic auditory neuropathies are limited to the auditory nerve. Most cases of nonsyndromic auditory neuropathy result from impaired synaptic transfer.^240^ Genetic auditory synaptopathies generally only cause deafness, such as mutations in the CACNA1D gene encoding the Ca_V_1.3L-type Ca2+ channel, the OTOF gene encoding Otoferlin, the SLC17A8 gene encoding Vglut3, and the DIAPH3 gene encoding formin 3.

Calcium influx at the base of the IHC near the synapse is mediated via the Ca_V_1.3L-type Ca2+ channel, encoded by the CACNA1D gene. Otoferlin, encoded by the OTOF gene, is a protein responsible for regulating the exocytosis of glutamatergic vesicles at the presynaptic site. The Vesicular Glutamate Transporter type 3 (VGLUT3 gene) is responsible for glutamate uptake at the postsynaptic site.^249^, ^250^

#### Otoferlin-DFNB9

The OTOF gene encodes otoferlin, which is a critical calcium sensor for exocytosis at the IHC synapse.^251^, ^252^ Mutations in the gene encoding otoferlin are responsible for profound autosomal recessive prelingual deafness, DFNB9.^253^ Approximately 220 pathogenic variants in OTOF have been identified.^254^ Patients with variants in OTOF have shown milder hearing loss as well as progressive and temperature-sensitive hearing loss, with preserved OAEs.^253^, ^255^, ^256^ Children harboring biallelic mutations in the OTOF gene have shown profound hearing loss and absence of ABRs and CAP, with preserved DPOAE and CM amplitude.^257^

Otoferlin knockout mice have profound deafness due to a failure of neurotransmitter release at the IHC synapse and are likely to be an appropriate animal model for DFNB9.^257^, ^258^ In these mice, exocytosis triggered by Ca2+ in IHCs is almost abolished.^259^ OTOF mutations are particularly prominent in the Spanish population, where up to 8% of autosomal recessive nonsyndromic hearing loss is due to these mutations.^256^ Available data from several case series show that CI outcomes for individuals with OTOF mutations are excellent and typically the same as for individuals with genetic mutations affecting the sensory partition.^256^, ^260^

#### DFNB59

Mutations in the DFNB59 gene are the second genetic cause of auditory neuropathy. The protein encoded by DFNB59 plays a role in the formation of peroxisomes that protect cells from damage during cellular antioxidant response. It is expressed in OHCs and IHCs as well as in spiral ganglion neurons. To date, there are 16 reported deafness-causing mutations in this gene, 2 of which are related to auditory neuropathy. There are no reports of CI outcomes in individuals with deafness caused by DFNB59 mutations.^240^, ^248^, ^261^

#### TBC1D24

Mutations in the TBC1D24 gene cause hearing loss, genetic epilepsy, onychodystrophy, mental retardation, and seizures. TBC1D24 mutations cause severe-to-profound congenital autosomal recessive nonsyndromic sensorineural hearing loss, in addition to progressive dominant nonsyndromic sensorineural hearing loss. The TBC1D24 gene encodes the Tre2-Bub2-Cdc16 protein expressed in the cilia of IHCs and OHCs and in the spiral ganglion. TBC1D24 mutations cause postsynaptic auditory synaptopathy.^248^

#### VGLUT3-DFNA25

Vesicular Glutamate Transporters (VGLUTs) are responsible for glutamate loading into synaptic vesicles, which is essential for achieving synaptic transmission. VGLUT3 is expressed in small subsets of neurons in the central nervous system.^262^ Genetic ablation of SLC17A8 in mice results in the absence of ABRs to acoustic stimuli, whereas ABRs can be captured by electrical stimuli, with robust OAEs being recorded in these mice.^263^ Therefore, this reflects a failure in the activation of the ascending auditory pathway, while the activity in OHCs is not affected.^263^ Patients with a 12q22-q24 deletion in the SLC17A8 gene at the DFNA25 locus have congenital autosomal dominant nonsyndromic deafness.^263^ Deafness in patients was characterized as progressive high-frequency sensorineural hearing loss, with good hearing rescue through CI, thus reinforcing the hypothesis of synaptopathy.^239^, ^263^

Calcium influx at the base of the IHC near the synapse is mediated via the Ca_V_1.3L-type Ca2+ channel, which is the main voltage-gated Ca2+ channel in IHCs and essential for hearing. Ca_V_1.3 translates sound-induced depolarization into neurotransmitter glutamate release at the synaptic site, resulting in signal transmission to the auditory nerve.^264^ Ca_V_1.3 encoded by the CACNA1D gene is widely distributed across different cells, such as OHCs, IHCs, cardiomyocytes, neuroendocrine cells, and neurons. Mutations in this gene can cause Sinoatrial Node Dysfunction and Deafness (SANDD) syndrome in mice and in humans.^264^, ^265^

#### CABP2-DFNB93

Calcium-Binding Protein 2 (CABP2) is a potent modulator of IHC Ca_V_1.3 voltage-gated calcium channels. CABP2 regulates Ca2+ influx at the presynaptic site as well as the vesicular release of glutamate.^266^ Pathologic mutations in CABP2 lead to moderate-to-severe autosomal recessive nonsyndromic hearing loss, DFNB93.^27^, ^267^ DFNB93 hearing impairment may result from an enhanced steady-state inactivation of Ca_V_1.3 channels at the IHC synapse, limiting their availability to trigger synaptic transmission, resulting in elevated auditory thresholds.^268^ This does not interfere with cochlear development and does not cause early degeneration of hair cells or their synaptic complex. These results suggest an extended window for gene therapy.^239^

#### DIAPH3-AUNA1

Nonsyndromic Autosomal Dominant Auditory Neuropathy 1 (AUNA1) is a form of late-onset progressive deafness resulting from a point mutation in the 5'untranslated region of the Diaphanous Homolog 3 (DIAPH3) gene. The DIAPH3 mutation leads to overexpression of the DIAPH3 protein, a member of the formin family involved in cytoskeletal dynamics.^269^ Morphologic evaluation revealed selective and early alteration of the IHC plaque and fused stereocilia with eventual loss of IHC ability to transmit sensory stimuli.^269^ In addition, a significant reduction in the number of IHC synapses was observed.^269^ Altogether, these results suggest an important function of Diap3 in regulating the assembly and/or maintenance of actin filaments in IHC stereocilia, as well as a potential role in the IHC synapse.

#### TMPRSS3

Mutations in the TMPRSS3 gene cause 2 types of autosomal recessive sensorineural hearing loss: severe-to-profound congenital deafness (DFNB10) and progressive postlingual Deafness (DFNB8). There are 41 reported TMPRSS3 mutations identified as causing deafness. This gene encodes the transmembrane serine protease 3 protein, expressed in IHCs and OHCs and in the spiral ganglion.^270^

### Syndromic auditory neuropathy

Genetic neuropathies often affect other neurons, leading to syndromic phenotypes such as Charcot-Marie-Tooth (CMT) disease, Autosomal Dominant Optic Atrophy (ADOA), Leber Hereditary Optic Neuropathy (LHON), Friedreich Ataxia (FRDA), Mohr-Tranebjaerg syndrome, Refsum disease, and Wolfram syndrome.

#### Charcot-Marie-Tooth

Autosomal dominant CMT is the most common hereditary peripheral polyneuropathy, characterized by peripheral nerve degeneration. CMT can be classified into 2 major categories: CMT type 1 (demyelinating neuropathies) and type 2 (axonal form of neuropathies).^271^ Patients with CMT carry mutations in the genes that encode proteins essential for the formation and adhesion of myelin.^245^, ^272^ CMT type 1 A (CMT1A) is the predominant subtype, which is a demyelinating peripheral neuropathy characterized by distal muscle weakness, sensory loss, areflexia, and slow motor and sensory nerve conduction velocities.^272^ Hearing impairment is also a relatively common symptom of CMT1A. Compared with controls, patients with CMT1A showed a significant decrease in speech discrimination in noisy environments, as well as a decrease in temporal and spectral resolution, suggesting that demyelination of auditory nerve fibers in CMT1A causes defective cochlear neurotransmission.^273^ Patients with CMT type 1 and 2 showed delayed or reduced ABR amplitude, as well as impaired speech intelligibility, which are electrophysiologic evidence of auditory neuropathy.^271^ These patients should be closely monitored. Depending on the hearing loss, a CI or hearing aid may be used.^274^

#### Friedreich ataxia

FRDA is the most common autosomal recessive hereditary ataxia caused by mutations in the FXN gene encoding the mitochondrial protein Frataxin, involved in the regulation of iron accumulation in mitochondria. In addition to impaired balance and coordination of voluntary movements, FRDA is associated with hearing impairment, including difficulty understanding speech in background noise, but, in most cases, pure-tone thresholds as well as OHC function remain unchanged. Only 8%–13% have sensorineural hearing loss, as revealed in a pure-tone audiogram. Most affected individuals show abnormalities in speech discrimination and ABRs as a result of auditory neuropathy.^275^, ^276^

#### Leber hereditary optic neuropathy

LHON is the most common mitochondrial genetic disease. It is characterized by bilateral, subacute, painless vision loss, and more than 95% of cases of LHON are caused by 1 of 3 mitochondrial DNA (mtDNA) point mutations: 3460 G > A, 11778 G > A, and 14484 T > C or mutation in the TMEM126A gene encoding a mitochondrial protein. Severe axonal degeneration with optic nerve demyelination has been indicated by histologic necropsy studies. Patients with LHON also show signs of auditory neuropathy.^277^

#### Mohr-Tranebjaerg syndrome

Mohr-Tranebjaerg syndrome, in which deafness with progressive dystonia and visual impairment are associated, can be classified as a non-isolated auditory neuropathy. In fact, post-mortem sample analysis demonstrates neuronal loss with preservation of OHCs. Mutations (DDP1 for deafness-dystonia) in *TIMM8A*/DDP1, gene encoding a 97-amino-acid polypeptide located in the mitochondria, are at the origin of this syndrome.^278^

#### Autosomal dominant optic atrophy

ADOA is the most common form of hereditary optic neuropathy, with a reported frequency of 1:10,000, and is caused by heterozygous variants in the OPA1 gene encoding a mitochondrial dynamin-related large GTPase. OPA1 is involved in several mitochondrial functions, notably in the maintenance of the respiratory chain and cell membrane potential, control of apoptosis, and mtDNA maintenance. ADOA was initially described as a moderate-to-severe nonsyndromic loss of visual acuity with insidious onset in early childhood caused by progressive loss of retinal ganglion cells. Over the past decade, the clinical spectrum of ADOA has been extended to a wide variety of symptoms, including deafness, ataxia, neuropathy, and myopathy, and is now called ADOA plus.^279^ Deafness is the second most prevalent clinical feature in ADOA plus, affecting approximately 20% of all patients with ADOA.^257^

Hearing loss begins in childhood or early adulthood. Although most studies broadly qualify the hearing disorder as ‘sensorineural hearing loss,’ some authors have proposed that auditory neuropathy is the pathophysiologic mechanism underlying the hearing impairment in OPA1-ADOA. The audiologic testing of hearing-impaired patients with OPA1 mutations showed impairment in speech perception and absence or profound alteration of ABRs but preservation of OAEs and even enhanced CM potentials, reflecting normal OHC function.^257^

#### Cowchock syndrome

Mutations in the AIFM1 gene cause neuropathy and are associated with Cowchock syndrome, a neuromuscular disorder associated with sensorineural hearing loss. It is an X-linked mutation where, in addition to the initial auditory neuropathy, individuals later develop sensory neuropathy with extremity paresthesias, unsteadiness, and areflexia. It is rare, and to date, there are few reports of AIFM1 mutations causing deafness.^280^

Variants in the AIFM1 gene are a common cause of familial and sporadic ANSD and provide insight into the expanded spectrum of AIFM1-related diseases. The finding of cochlear nerve hypoplasia in some patients was consistent with AIFM1-related ANSD, implying that MRI may be useful in locating the lesion site and suggesting that CI in these patients may have limited success.^281^

#### Alpers or Leigh syndrome

While NARS2 is widely expressed in human tissues, variants in the gene appear to preferentially affect tissues with high energy demand, such as the brain, cochlea, and muscle, similar to other mitochondrial disorders.^282^ Homozygous mutations in NARS2 have been associated with Combined Oxidative Phosphorylation Deficiency 24 (COXPD24) and autosomal recessive Deafness 94 (DFNB94). COXPD24 is an autosomal recessive mitochondrial disorder that often manifests early in life with seizures, hypotonia, myopathy, hearing impairment, and overall delay and/or regression of cognitive and motor development. CT and MRI may reveal bilateral brain lesions or diffuse progressive cerebral atrophy indicative of Alpers or Leigh syndrome.^283^ DFNB94 is characterized by bilateral nonsyndromic sensorineural hearing loss because the NARS2 protein is expressed in the spiral ganglion and other cells of the organ of Corti.^31^

#### Hearing rehabilitation in ANSD

Some studies show varying results with CI in 25% of cases, which are dependent on other patient conditions such as the presence of residual hearing, age at the time of CI surgery, duration of auditory deprivation before CI, aspects of the patient’s cognition, socioeconomic factors, and anatomic morphology, mainly of the cochlea and cochlear nerve.^1^, ^51^, ^248^, ^284^ Patients with ANSD have a huge advantage with CIs than with PSAPs, but they have poorer responses in open-set speech perception than implanted patients with pure-tone cochlear etiologies for sensorineural hearing loss.^285^

CI users experience significant improvement in speech perception and auditory performance. Even those with the worst results show a 50% improvement in the recognition of disyllabic words in audiologic tests after CI, in addition to greater ease of phone use and improved speech understanding in background noise, especially for adults.^286^, ^287^

The CI may provide positive results even when the lesion site seems to involve the auditory nerve. Molecular markers can be of great assistance when making the final treatment decision. In addition to the use of hearing aids, auditory training should focus on maximizing the signal-to-noise ratio – improving speech perception in noise with or without the use of a frequency modulation system or similar device – always providing appropriate counseling and defining the limitations that are more closely related to postsynaptic or central nervous system lesions.

CI candidates show variations in auditory nerve function when responding to CI electrical stimulation. Assessing the integrity of the spiral ganglion and cochlear nerve has become an important focus in predicting CI outcomes. Several studies have attempted to determine whether CI will benefit individuals with auditory neuropathy. Auditory neuropathy was initially considered a contraindication to CI. However, after initial reports of successful CI in these patients, larger studies were conducted and observed that patients with ANSD may benefit from CI depending on the lesion site (whether having neuropathy or synaptopathy).^248^, ^280^

Determining the precise site of lesion causing neuropathy – by molecular diagnosis combined with electrophysiologic data from ECochG as well as with the use of intraoperative electric ABR (EABR) – may help predict postoperative CI outcomes and may assist in the final treatment decision.^288^, ^289^ Due to the extreme genetic heterogeneity of deafness, adequate studies require a large number of patients. Therefore, any study designed to report outcomes for CI users with ANSD should include:

  1) A detailed medical history of the patient, with family history of neuropathy, vision loss, or vestibular deficits;
  2) Audiologic data on the degree of hearing loss, age of onset of hearing loss, and speech recognition scores in quiet and in noise;
  3) Electrophysiologic data from OAE, ABR, EABR, and ECochG (CM, summating potential, auditory nerve neurophonic potential, and adaptation);
  4) CI device implanted and type of stimulation;
  5) Post-implant outcome measures including word recognition scores in quiet and in noise;
  6) Genetic/molecular data.

Another complicating factor is that the OAE response diminishes and disappears over time in approximately 20%–30% of individuals.^290^ Given the limitations imposed on NHS programs, molecular genetic testing has gained importance in the screening of newborns, children, and adults who are CI candidates. Several deafness-causing genes have known effects on the midbrain and auditory cortex, including DFNB59, CACNA1D, and KCNQ4.^291^

#### Gene therapy

Gene therapy treats genetic diseases by manipulating target genes via multiple methods including gene replacement, gene suppression, and gene editing strategies. Gene replacement, a dominant strategy in clinical studies, provides sufficiently functional protein by delivering exogenous genes, and this method is suitable for the treatment of both recessive and dominant genetic diseases that have insufficient single allele dosage, such as OTOF mutation-induced autosomal recessive DFNB9.^239^

Adeno-Associated Virus (AAV)‐based gene therapy has become a promising potential treatment to reverse OTOF mutation‐induced deafness.^292^ Between October 19, 2022, and June 9, 2023, Lv et al.^293^ screened 425 participants for eligibility and enrolled 6 children for AAV1-hOTOF gene therapy. Five children had hearing recovery, shown by a 40–57 dB reduction in the average ABR thresholds at 0.5–4.0 kHz. In the participant who received the highest dose of medication, the average ABR threshold improved from 95 dB at baseline to 68 dB at 4 weeks, 53 dB at 13 weeks, and 45 dB at 26 weeks. In those who received lower doses, the average ABR thresholds changed from greater than 95 dB at baseline to 48 dB, 38 dB, 40 dB, and 55 dB in 4 children with hearing recovery at 26 weeks. Speech perception was improved in participants who had hearing recovery.

#### Recommendations

LIII Even for patients with mutations affecting the auditory nerve, CI can still be recommended due to better hearing outcomes for most patients. (Strong Recommendation – Moderate-Quality Evidence).
LIV Lesion site is not the only determinant of CI outcome. Not only clinical factors, such as type and duration of deafness, should be considered, but also molecular and physiologic factors that may suggest the status of the cochlea, synapse, spiral ganglion, and auditory nerve to provide the best outcomes for patients. (Strong Recommendation – Low-Quality Evidence).
LV Despite the success of gene therapy in preliminary studies of patients with mutations in the OTOF gene, CI is still indicated for this group of patients, especially considering, until the publication of this article, the unavailability of this therapy for the general population. (Strong Recommendation – Moderate-Quality Evidence).
LVI The possibility of performing surgery on only one ear can be discussed with the family of patients with auditory neuropathy to rehabilitate and await future gene therapy. However, it is necessary to properly monitor the patient for surgery on the contralateral ear if the child does not develop adequate language with only one ear implanted. (Weak Recommendation – Low-Quality Evidence).

### Inner ear malformations

Congenital hearing loss is mostly caused (80%) by membranous malformations involving the hair cells of the inner ear, without macroscopic bony abnormality. Therefore, these cases show normal findings on high-resolution CT and MRI of the temporal bone. The remaining 20% show a wide variety of malformations involving the bony labyrinth and, therefore, can be demonstrated radiologically.^294^ The majority of these patients have severe-to-profound bilateral hearing loss and are candidates for CI surgery. Some severe malformations may require a different surgical approach in CI surgery, such as performing subtotal petrosectomy. There is a wide variety of classifications for these malformations, but the most used is the one proposed by Sennaroglu et al.^295^

Three factors should be considered before making a preoperative decision to indicate CI, selecting the type of electrode array, and choosing the most appropriate technique: classification of the malformation, presence of a cochlear nerve, and preoperative audiologic test results. The main challenges for CI surgery in patients with inner ear malformations are gusher, risk of meningitis, and facial nerve anomalies.

#### Malformations in which cochlear implant is contraindicated

CI is contraindicated in the most severe malformations, with Auditory Brainstem Implant (ABI) being the only option for auditory rehabilitation.^296^

**Michel deformity (labyrinthine aplasia)**^294^, ^297^, ^298^ - Absence of the cochlea, vestibule, semicircular canals, and vestibular and cochlear aqueducts. The IAC consists only of the facial canal, and the labyrinthine, tympanic, and mastoid segments of the facial nerve can be identified in the temporal bone. Development of middle ear ossicles is generally normal.

**Rudimentary otocyst**^295^, ^298^ - Otic capsule with incomplete development, generally round or ovoid in shape, without communication with the IAC. Small appendages may develop on the otocyst, which may be a rudimentary labyrinth.

**Cochlear aplasia**^295^, ^298^ - Absence of the cochlea. The labyrinthine segment of the facial nerve is displaced anteriorly and occupies the normal location of the cochlea. The vestibule and semicircular canals are in their normal anatomic location: in the posterolateral part of the IAC. There are 2 subgroups according to the accompanying vestibular system:

A Cochlear aplasia with normal labyrinth: Vestibule and semicircular canals are normally developed.
B Cochlear aplasia with dilated vestibule: Vestibule and semicircular canals are dilated. It is very important to differentiate this condition from CC deformity. In cochlear aplasia with dilated vestibule, the IAC is normally developed, and the dilated vestibule occupies a normal location in the posterolateral part of the fundus. In CC deformity, the IAC enters the cavity at its center. In some patients, it may be very difficult to distinguish between these entities.

**Cochlear hypoplasia type I (bud-like cochlea)**^1^, ^2^ - The cochlea is a small bud, round or ovoid in shape, arising from the IAC. The internal architecture is severely deformed. Modiolus and interscalar septa cannot be identified.

Milder anomalies, such as incomplete partitions and EVA, allow good performance with CI,^297^, ^299^ but patients with more severe anomalies, such as CC deformity, are less likely to achieve significant speech development.^300^, ^301^

**Common cavity deformity**^297^, ^298^, ^302^, ^303^ - CC is a single, ovoid or round chamber representing the cochlea and vestibule. It has cochlear and vestibular neural structures in its wall. It may have semicircular canals or their rudimentary parts. The IAC usually enters the cavity at its center. The differential diagnosis is cochlear aplasia with dilated vestibule. A CI can be used, but usually the outcomes are not completely adequate. Perimodiolar electrodes are contraindicated because the neurons are located on the periphery of the cavity rather than in its center. There is an increased risk of migration of the electrode array into the IAC or even injury to the carotid canal.^304^

**Cochlear hypoplasia -** In cochlear hypoplasia and incomplete partitions, there is a clear differentiation between the cochlea and vestibule. Cochlear hypoplasia represents a group of malformations in which the external dimensions are smaller than those of a normal cochlea with various internal architectural deformities. In the smaller cochlea, it is often difficult to count the number of turns with CT and/or MRI. However, the definition ‘cochlea with 1.5 turns’ should be used for hypoplasia (particularly type III) rather than for incomplete partition type II.

Treating patients with cochlear hypoplasia can be challenging. Most patients have severe-to-profound hearing loss.^305^ However, they may present with different thresholds on audiometry, such as mild-profound sensorineural, conductive, or mixed hearing loss.^305^, ^306^ Decision-making about amplification options can be difficult, particularly in patients with a hypoplastic cochlear nerve. During CI surgery, facial nerve malposition should be expected due to associated semicircular canal abnormalities (particularly of the lateral canal).^307^ In the hypoplastic cochlea, the promontory may lack the usual protuberance and it may be difficult to identify the promontory and RW through the facial recess.^308^ In these situations, an additional transcanal approach may be required to expose the hypoplastic cochlea.

Cochlear Nerve Deficiency (CND) is often observed in patients with cochlear hypoplasia. The best option in these cases is to perform CI on the side with the better developed cochlear nerve or better audiologic findings. If there is limited hearing and language development with CI, an ABI should be considered.^296^

Four different types of cochlear hypoplasia have been defined:^294^, ^295^, ^298^

Type I (bud-like cochlea) ‒ Cochlear hypoplasia type I was described earlier.

Type II (hypoplastic cochlea) – The cochlea is smaller in size with defective modiolus and interscalar septa, but with normal external outline. There may be complete absence of modiolus, creating a wide connection with the IAC, thus making gusher and CI electrode migration into the IAC possible. The vestibular aqueduct may be enlarged, and the vestibule may be dilated. Recurrent meningitis may occur because of defective stapes footplate.

Type III (cochlea with less than 2 turns) – The cochlea has fewer turns (less than 2 turns) with a short modiolus. The overall length of the interscalar septa is reduced. The internal (modiolus, interscalar septa) and external outline are similar to that of a normal cochlea, with fewer turns and smaller dimensions. Vestibule and semicircular canals are usually hypoplastic. The cochlear aperture may be hypoplastic or aplastic. Hearing loss can be sensorineural, conductive, or mixed due to stapedial fixation.

Type IV (cochlea with hypoplastic middle and apical turns) – The cochlea has a normal basal turn, but the middle and apical turns are severely hypoplastic and located anteriorly and medially rather than in their normal central position. The labyrinthine segment of the facial nerve is usually located anterior to the cochlea rather than in its normal location. Hearing loss can be sensorineural, conductive, or mixed due to stapedial fixation. Treatment includes PSAPs, stapedotomy, and CI depending on the results of the evaluation.

#### Incomplete partition

Incomplete partitions represent a group of cochlear malformations where there is a clear differentiation between cochlea and vestibule, with normal external dimensions and several internal architectural defects. Incomplete partitions account for 41% of inner ear malformations.^298^ There are 3 different types of incomplete partition groups according to the defect in the modiolus and interscalar septa.

**Incomplete partition type I (IP-I) (cystic cochleovestibular malformation)**^302^, ^309^, ^310^ - They account for approximately 20% of inner ear malformations. The cochlea is located in its usual location in the anterolateral part of the fundus of the IAC and lacks the modiolus and interscalar septa (empty cystic structure). External dimensions (height and length) of an IP-I cochlea are similar to normal cases. The cochlea is accompanied by an enlarged, dilated vestibule. There may be a defect between the IAC and the cochlea due to abnormal development of the cochlear aperture and absence of the modiolus, and the CSF may completely fill the cochlea.

Patients are at increased risk of recurrent meningitis and spontaneous CSF leak due to defective stapes footplate, which allows the development of a cyst that facilitates communication with the middle ear. Most patients with IP-I have severe-to-profound sensorineural hearing loss. They are almost always candidates for CI, but there is an increased risk of gusher. There is also an increased risk of cochlear nerve aplasia, and ABI is indicated in this case.

**Incomplete partition type II (IP-II)**^297^, ^302^, ^311^ - The apical part of the modiolus and the corresponding interscalar septa are defective, giving the apex of the cochlea a cystic appearance due to the confluence of middle and apical turns. Patients do not have a characteristic hearing level, as their audiometric threshold test varies from normal to profound. Hearing loss is usually progressive and fluctuating. It can also be sudden (especially in patients who have suffered a traumatic brain injury, even a mild one). An air-bone gap may occur, particularly at low frequencies, due to a “third window” effect of the EVA and may resemble the audiometric findings of superior canal dehiscence syndrome. In some cases, this may mislead the surgeon to recommend stapedotomy. In cases of CI, there is an increased risk of fistula and gusher.

This anomaly was originally described by Carlo Mondini and, together with a minimally dilated vestibule and an EVA, they constitute the triad of Mondini deformity. The term “Mondini” should only be used if the aforementioned triad of malformations is present.^295^, ^302^, ^312^ The external dimensions of the cochlea (height and diameter) are similar to those observed in normal cases.^313^ Therefore, it is not correct to define this anomaly as a cochlea with 1.5 turns, and this term should only be used for cochlear hypoplasia type III.

**Incomplete partition type III (IP-III)**^297^, ^298^, ^302^, ^314^^,^^315^ - The cochlea has interscalar septa, but the modiolus is completely absent. IP-III cochlear malformation is the type of anomaly present in X-linked deafness. It is the rarest form of incomplete partition cases (about 2%). The otic capsule surrounding the membranous labyrinth is thinner than that in a normal cochlea. Instead of the usual 3 layers, the second and third layers are probably missing or very thin. The innermost endosteal layer appears to be thickened without the endochondral and outer periosteal layers. The labyrinthine segment of the facial nerve is located almost above the cochlea, rather than making a gentle curve around the basal turn on axial sections.

Hearing loss can be mixed or moderate-to-profound sensorineural. The conductive component may be due to the thin otic capsule and may resemble stapedial fixation. Stapes surgery is contraindicated in this group as it may lead to gusher and worsening of hearing thresholds. Patients with moderate-to-severe mixed or sensorineural hearing loss can be treated with hearing aids. Patients with profound hearing loss are candidates for CI. Given the absent modiolus and defect at the cochlear base, all patients with IP-III have severe gusher during CI surgery and there is a very high likelihood of electrode migration into the IAC. The electrode position should be confirmed intraoperatively in all cases of IP-III. Perimodiolar electrodes should be avoided. Spontaneous CSF leak through the stapes footplate and recurrent meningitis are very rare in IP-III despite high-volume CSF leakage during CI surgery. This is likely due to normal endosteal development (hence a normal footplate) in IP-III. IP-III cases have normal cochlear nerves. Therefore, ABI is not indicated in this group of incomplete partitions.

#### Enlarged vestibular aqueduct

EVA can be observed in nonsyndromic hearing loss if there is homozygosity for wild-type SLC26A4 or only one mutated allele.^316^ Pendred syndrome accounts for up to 10% of hereditary hearing loss, with an incidence of 7.5–10 per 100,000 population.^317^ First described in the literature by Pendred in 1896, it is characterized by sensorineural hearing loss, inner ear malformations (IP-II and EVA), and thyroid dysfunction.^318^ Pendred syndrome is inherited in an autosomal recessive manner and results from a biallelic mutation in the PDS/SLC26A4 gene.^319^

Two other genes have been described to cause Pendred syndrome, accounting for less than 2% of affected individuals: FOXI1 encoding Forkhead box protein I1 and KCNJ10 encoding the adenosine triphosphate-sensitive potassium channel.^320^ Individuals with Pendred syndrome or DFNB4 should undergo annual hearing testing due to the possibility of progressive hearing loss. In addition, thyroid function should also be monitored in children with Pendred syndrome, as there is a risk of developing goiter during puberty.^321^

Audiologic phenotypes may vary, with mild-to-profound, congenital, or late-onset hearing loss. Most patients present with severe-to-profound progressive congenital hearing loss, which can be aggravated by traumatic brain injury or barotrauma.^321^ Especially in children, the only clue to the diagnosis of Pendred syndrome may be the detection of radiologic abnormalities – EVA and IP-II. These diagnoses have important implications for management, as children with EVA may present with sudden and significant hearing loss after mild traumatic brain injury.^322^

Goiter is characteristically multinodular, with onset in the second decade of life. It develops during puberty in 40% of cases and in adulthood in 60%.^317^ Thyroid hormones can be at normal (in most cases) or low levels. Delayed thyroid iodine organification results in a positive perchlorate discharge test.^320^

#### Cochlear nerve deficiency

Cochlear nerve aplasia is the absence of a cochlear nerve, while cochlear nerve hypoplasia is defined as the presence of a cochlear nerve with a caliber smaller than that of the remaining nerves seen within the IAC, mainly the facial nerve, in the parasagittal plane.^298^, ^323^ Patients with cochlear nerve hypoplasia or aplasia, known as CND, often present with associated labyrinthine abnormalities, with widely varying degrees of severity.^324^ Children with CND have severe-to-profound sensorineural hearing loss.

CND, one of the main anomalies of the inner ear, is found in approximately 1%–5.3% of children with bilateral sensorineural hearing loss.^325^, ^326^ The possibility of the presence of a cochlear nerve fiber should be considered even when preoperative evaluation points to apparent cochlear nerve aplasia. Correspondingly, evidence of CND as a potential indication for CI has increased.^325^, ^326^

High-resolution CT of the temporal bone can be useful in assessing the health of the cochlear nerve. The cochlear nerve is considered hypoplastic or aplastic when the diameter of the bony cochlear nerve canal is <1.5 mm and the diameter of the IAC is <2 mm.^327^, ^328^

As the indications for CI have broadened, more patients with cochlear nerve aplasia and hypoplasia have undergone CI with varying results. Furthermore, MRI in the axial and sagittal planes is routinely performed in children with sensorineural hearing loss, which has allowed for accurate determination of cochlear nerve aplasia vs hypoplasia.

Some authors have reported that patients with cochlear nerve hypoplasia or aplasia are able to achieve sound detection with CI.^329^, ^330^, ^331^ The ability of patients with cochlear nerve aplasia to experience auditory stimulation from a CI is likely due to the cochlear nerve fibers traveling with other nerves in the IAC, a hypothesis that has been supported by anatomic studies.^332^, ^333^

The morphology of the cochlear nerve or vestibulocochlear nerve and the width of the cochlear nerve canal may predict the degree of CND. Patients with aplastic cochlear nerves tend to perform worse than those with hypoplastic cochlear nerves after CI.^331^, ^334^ Furthermore, the width of the IAC is positively correlated with the diameter of the cochlear nerve, and a narrower bony cochlear nerve canal has been associated with more severe hearing loss and lower speech discrimination scores than a wider canal.^335^ Therefore, the size of the cochlear nerve and the width of the IAC on imaging may be indicators of the severity of CND, but there is no consensus in the literature.^336^, ^337^

Although the integrity of the cochlear nerve is a critical factor affecting the postoperative effects of CI, age at implantation, history of hearing aid use, cognitive ability, parental socioeconomic status, and language training are also factors that contribute to CI surgery outcomes. Therefore, the relationships between preoperative imaging findings and cochlear nerve function remain unclear.

ECAPs can be used to assess cochlear nerve function in CI users.^338^ The ECAP response is generated by a group of auditory nerve fibers that are activated by electrical stimuli. It may be recorded using the ‘reverse’ telemetry function implemented in current CI devices. Previous studies have shown that the slope of the ECAP Input/Output (I/O) function and the ECAP amplitude evoked by the most comfortable level are associated with the density of the surviving neural population, with steeper slopes and larger amplitudes suggesting a larger number of residual neurons.^339^, ^340^

Furthermore, the ECAP threshold, which refers to the lowest stimulation level that could evoke an ECAP response, may also reflect the neural population to some extent.^341^ However, poor responsiveness and the possibility of electrical artifacts between electrodes and cochlear nerve fibers make it difficult to use ECAP in patients with cochlear nerve aplasia. Studies such as that by Yamazaki et al.^342^ have shown that intracochlear EABR, along with cochlear nerve integrity on MRI, is clinically significant in predicting postoperative CI outcomes. EABR has been shown to be more sensitive than ECAP. If the MRI and intraoperative EABR testing demonstrate ‘CN7 > Cn8/negative eV’ on both sides, it is reasonable to consider ABI because it is the only way to provide effective auditory sensation, and the critical period for auditory development should not be missed.^342^

Children with cochlear nerve aplasia are less likely to benefit from CI than those with cochlear nerve hypoplasia.^343^, ^344^ However, variable CI outcomes occur in children with cochlear nerve aplasia, resulting in a range of auditory performance from awareness of environmental sounds to conversation without lip reading, implying that a subgroup of patients with cochlear nerve aplasia may achieve closed- or open-set levels of speech perception after CI.^344^ Considering the unpredictable outcomes of CI in patients with cochlear nerve aplasia, it may be inappropriate to apply the same rehabilitation strategy to all patients and CI should not necessarily be pursued and maintained longitudinally. In fact, some studies have shown that ABI improves hearing in children with cochlear nerve aplasia who had unsuccessful bilateral CI operations,^344^ with better auditory perception and language development outcomes in the pediatric ABI group than in the CI group.^343^

Yousef et al.^343^ compared and analyzed auditory performance after implantation in 14 patients with CND. Of these, 7 received a CI and 7 received an ABI. Five patients in the CI group and all patients in the ABI group had bilateral cochlear nerve aplasia. The mean Categories of Auditory Performance (CAP) score at 2 years after implantation was 1.29 in the CI group and 2.87 in the ABI group, demonstrating better outcomes in the ABI group. However, not all studies in the literature support this probably due to small sample sizes for statistical significance, confounding factors, or heterogeneous assessment of auditory and speech performance in patients with cochlear nerve aplasia.

A meta-analysis demonstrated that, among pediatric patients with CND, 25% (27/108) achieved open-set speech perception and 34% (37/108) achieved closed-set speech perception after CI,^344^ suggesting that CI may serve as an initial treatment before ABI in children with CND. The rationale behind this could be the presence of residual cochlear nerve fibers that were too hypoplastic to appear on MRI, even when cochlear nerve aplasia was documented. A subset of children with CHARGE syndrome benefit from CI because residual cochlear nerve fibers exist, although they are very small and follow an alternative course. Furthermore, children with apparent cochlear nerve aplasia on MRI could benefit from electrical stimulation to develop auditory performance, which is likely due to connections or anastomoses between the cochlear nerve and the adjacent vestibular nerve based on anatomic studies.^332^, ^333^ Choe et al.^345^ suggest that CI may be useful as an initial treatment before ABI in children with cochlear nerve aplasia, as evidenced by the result that 57.1% (12/21) obtained a CAP score ≥3 at 3 years post-CI.

#### Facial nerve

Because the facial nerve develops closely with the inner ear, abnormalities in its normal anatomic course may coincide with premature embryologic arrest and are an important perioperative consideration for the surgeon when choosing the surgical approach, as the nerve may prevent traditional access to the RW. A systematic review found facial nerve anomalies in 34.4% of cases of inner ear malformations,^297^ which is considerably higher than the 0.3% prevalence rate in normal ears.^346^ CC deformity, cochlear hypoplasia, and IP-I are associated with a more frequent abnormal course of the nerve. Imaging does not always rule out an anomaly, so it is important to keep this in mind when operating on higher risk patients to avoid unexpected injury. In some cases, a retrofacial approach to access the posterior mesotympanum and visualize the RW may be helpful, but this approach predisposes the patient to facial nerve injury during surgery.

#### Gusher

CSF gusher occurs with opening of the cochlea (via RW or cochleostomy) in some patients. It increases the risk of developing postoperative meningitis. According to Phelps et al.,^347^ CSF ooze is described as a flow of fluid in small quantities, while gusher denotes a profuse flow. This is typically resolved by elevating the patient’s head to decrease intracranial pressure and by obliterating the cochleostomy/RW. Some special types of electrodes have also been studied to help control gusher. CC malformations, IP-II, and IP-III, in addition to EVA, have an increased association of lateral wall anomalies of the IAC and, therefore, an increased risk of gusher.

#### Recommendations

LVII CI should not be indicated for patients with the following inner ear malformations: Michel deformity; rudimentary otocyst; cochlear hypoplasia type I; and cochlear aplasia. (Moderate Recommendation – Low-Quality Evidence).
LVIII Patients with CC deformity and IP-III have an increased risk of migration of the electrode array into the IAC, making intraoperative imaging essential before neurotelemetry. (Moderate Recommendation – Low-Quality Evidence).
LIX Patients with EVA should be referred for clinical and genetic evaluation for the diagnosis of Pendred syndrome. (Moderate Recommendation – Moderate-Quality Evidence).
LX Patients with CC malformations, IP-II, and IP-III, in addition to EVA, have an increased association of lateral wall anomalies of the IAC and, therefore, an increased risk of gusher; thus, the surgeon should be prepared to adequately control this complication. (Strong Recommendation – Low-Quality Evidence).
LXI CI surgery may be indicated to children with suspected cochlear nerve hypoplasia or aplasia as long as the family’s expectations are adequately met. (Moderate Recommendation – Moderate-Quality Evidence).
LXII Children with suspected unilateral cochlear nerve hypoplasia or aplasia and with a normal contralateral ear should not undergo bilateral CI surgery. (Moderate Recommendation – Moderate-Quality Evidence).
LXIII In children with cochlear nerve aplasia with no functional outcomes from CI surgery, ABI may be indicated. (Moderate Recommendation – Moderate-Quality Evidence).

### Cochlear ossification

There are few settings where the cochlea can be obstructed with ossified tissues that make electrode insertion challenging. Cochlear ossification often occurs secondary to meningitis, although advanced otosclerosis and other diseases can induce a similar process.^348^, ^349^ In this setting, efficient workup and surgical scheduling is necessary because ossification can progress rapidly and limit electrode insertion.

Ossification after bacterial meningitis presumably occurs by the spread of infection to the cochlea via the cochlear aqueduct.^349^ The inflammatory reaction within the cochlea progresses through several phases that begin as fibrosis and culminate in ossification that has its most dramatic effect on the basal turn of the cochlea, although the entire cochlea can be affected.^350^

In advanced otosclerosis, ossification occurs in the RW and extends to the basal turn of the cochlea.^351^ In most cases, ossification does not extend beyond the basal turn of the cochlea. To overcome the ossification encountered, several drilling techniques have been described that can be performed with the standard posterior tympanotomy approach.^352^ If access for drilling the ossified portion of the cochlea is limited, obliteration of the cavity and the canal wall has been described.^353^

Approximately 10% of patients with otosclerosis and conductive hearing loss also develop sensorineural hearing loss.^354^ Advanced otosclerosis is characterized by sensorineural hearing loss and decreased speech discrimination (<30% at 70 dB),^355^ associated with radiologic abnormalities.^356^ Sensorineural hearing loss in patients with otosclerosis occurs when ionic homeostasis of the cochlea is disrupted due to atrophy and hyalinization in the stria vascularis and spiral ligament. Consequently, dysfunction or loss of hair cells and loss of spiral ganglion can occur.^357^

The severity of sensorineural hearing loss in otosclerosis is correlated with radiologic abnormalities on high-resolution CT, which can detect oval window abnormalities in 80%–90% of cases.^358^, ^359^ On CT, the finding of pericochlear lucencies is highly specific for otosclerosis. It presents as a double halo.^360^, ^362^ T1-weighted MRI images may show a ring of intermediate signal in the pericochlear area with mild-to-moderate gadolinium enhancement.^362^ T2-weighted sequences are the best method to assess the patency of the cochlear duct.^358^, ^363^, ^364^

Intraoperative difficulties include ossification, partial obliteration of the basal turn and RW, and false tract insertion of electrode array into the cochlea.^351^ In a case series of advanced otosclerosis treated with CI surgery, the RW membrane was ossified in 60% of cases and the ST in 30% of cases.^365^ Some software programs have been developed by CI manufacturers with the purpose of conducting a detailed planning of the surgery as well as choosing the best electrode insertion method and the best electrode array system based on high-resolution CT data.^366^, ^367^

Facial nerve stimulation can occur both at the time of electrode activation and during subsequent device monitoring visits.^368^ To eliminate or at least minimize its effects, mapping adjustments should be done, such as decreasing the electric charges by changing the stimulation mode – by reducing the amplitude of the maximum current levels, keeping them below the stimulation threshold for the facial nerve, or even by adjusting the biphasic pulse width.^369^ More recently, triphasic pulse patterns have also been successfully used to alleviate the symptoms of facial nerve stimulation.^370^, ^371^

Switching off the problematic electrodes has been another procedure frequently described in studies as an alternative method to manage facial nerve stimulation.^372^ There is no consensus on which electrodes (basal, medial, or apical turn electrodes) are the most affected in facial nerve stimulation.^361^, ^373^ The fact is that, depending on the number of deactivated electrodes, speech perception can be significantly affected. In these cases, reoperation using another CI model with characteristics suitable for targeted electrical stimulation (related to the positions of intracochlear electrode contacts, electrode geometry, and stimulation parameters) as well as sequential CI may be viable alternatives to be considered.^369^, ^373^, ^374^

Most studies point to promising auditory perception outcomes in patients with advanced otosclerosis. Numerous studies have not found significant differences in word and speech recognition performance scores between implanted patients with advanced otosclerosis and those with other etiologies.^368^, ^369^, ^374^ In fact, it is assumed that this disease has little effect on the spiral ganglions of the auditory nerve.^366^ However, although not significant, some authors have reported a trend toward poorer performance in the group with advanced otosclerosis. The poorer results obtained in patients with advanced otosclerosis were associated with factors such as long deafness periods, older age, extensive ossification, presence of facial nerve stimulation, and a greater number of deactivated electrodes.^368^, ^375^, ^376^

The progression of otosclerotic foci often occurs in the basal and medial regions of the cochlea and, due to decreased impedance of the otic capsule and flow of the electric current through the bone, the electric current required to stimulate the fibers of the auditory nerve is increased. Mapping adjustments are essential to manage this situation. With the increase in stimulation levels, if the perceived intensity is not adequate, there is a need to increase pulse duration, which can potentially result in a decrease in stimulation frequency and compromise the proper functioning of the selected processing strategy. In certain situations, there may be a need to switch off the electrodes to avoid the negative effects generated by the significant increase in stimulation levels.^359^, ^369^, ^371^

Facial nerve stimulation resulting from a shunt of current from the otic capsule that reaches the labyrinthine segment of the facial nerve^362^, ^371^, ^376^ has been described as one of the most common postoperative complications in patients with advanced otosclerosis, with an average incidence of 20% in this population, reaching up to 75%.^361^, ^373^, ^374^ Authors have suggested that the high incidence of facial nerve stimulation is associated with the type of electrode array used (straight or perimodiolar), with the straight or more distal array showing a higher incidence.^359^, ^361^, ^373^, ^375^

Other techniques describing scala vestibuli insertion^377^, ^378^ and retrograde electrode insertion have been reported.^379^ Surgically, in addition to being challenging, ossification in the setting of meningitis and otosclerosis has higher reported rates of facial nerve stimulation than in traditional CI.^348^ There is also evidence demonstrating that patients with cochlear ossification who undergo CI often experience limited hearing benefit,^380^ given the reduction of spiral ganglion neurons due to ossification.^349^

Varying degrees of cochlear ossification may occur in more than 10% of all CI candidates.^381^ Diseases implicated in cochlear ossification include meningitis (bacterial),^348^, ^382^, ^383^ otitis media,^382^, ^384^ otosclerosis,^351^, ^384^ and autoimmune disease (Cogan syndrome).^384^, ^385^, ^386^ Several disorders such as temporal bone trauma, labyrinthine artery occlusion, temporal bone tumors, and Wegener’s granulomatosis are also associated.^348^, ^387^

The basal turn is the site most affected by most diseases. Two types of ossification have been described: metaplastic and osteoplastic.^388^ The metaplastic form (meningitis and otitis media) has high cellularity, low osteoblasts, and ill-defined margins. It is confined to the cochlear lumen with preservation of the endosteum. The osteoplastic form causes disruption of the endosteum (trauma and otosclerosis), leading to new bone formation that is lamellar, less cellular, with poorly defined margins.^388^

Bacterial meningitis is the most common cause of cochlear ossification reported in the literature.^383^ Infection reaches the cochlea through the cochlear aqueduct that communicates with the subarachnoid space.^389^ Spread to the cochlea is rapid and may occur within a few hours of the diagnosis of infection. The most affected region is the ST, reaching the organ of Corti posteriorly.^349^ *S. pneumoniae* is the most harmful agent.^389^ Meningitis has been associated with negative post-implantation speech outcomes, regardless of ossification or extent of electrode insertion.^390^, ^391^

Cochlear ossification remains a challenge in CI due to considerable changes in surgical techniques ranging from different surgical approaches (posterior tympanotomy, middle fossa, and subtotal petrosectomy),^348^, ^352^, ^387^ choice of electrode arrays (standard, compressed, and double),^352^, ^383^, ^392^, ^393^ and extent of drilling (RW, basal turn, middle turn/circum-modiolar)^348^, ^352^, ^387^, ^392^ to location and extent of electrode insertion (ST and scala vestibuli) (partial and complete).^382^, ^387^, ^393^, ^394^

Postoperative hearing outcomes are variable due to extent of electrode insertion, reduction in spiral ganglion cells,^382^, ^383^, ^384^, ^388^ increased impedance,^395^ and increased risk of electrode migration.^396^ Preoperative radiographic evaluation is essential for preoperative planning. CT and MRI are complementary modalities. The technique to be used varies depending on the degree of cochlear ossification.

#### Recommendations

LXIV MRI is essential for patients with suspected cochlear ossification. Gadolinium should be used in patients with a recent history of meningitis, particularly bacterial meningitis. (Strong Recommendation – High-Quality Evidence).
LXV Patients with signs of complete cochlear ossification on T2-weighted MRI sequences should not undergo CI surgery. (Strong Recommendation – Moderate-Quality Evidence).
LXVI The presence of ossification of the basal turn does not contraindicate CI surgery. (Moderate Recommendation – Low-Quality Evidence).
LXVII CI is safe and beneficial in cases of advanced otosclerosis. (Strong Recommendation – High-Quality Evidence).
LXVIII Patients with advanced otosclerosis are at increased risk of ossification of the RW membrane and basal turn, and the surgeon should order MRI as a mandatory test to prevent complications during the insertion of electrode array. (Strong Recommendation – Moderate-Quality Evidence).
LXIX Remodeling of the otic capsule in otosclerosis alters the properties of electric current conduction in the cochlea, which may impair CI use over time. More frequent mapping aimed at adjusting and optimizing stimulation parameters is recommended due to increased electrode impedance and risk of facial nerve stimulation. (Strong Recommendation – Moderate-Quality Evidence).
LXX The use of perimodiolar electrodes reduces the risk of facial nerve stimulation compared with lateral wall electrodes. (Moderate Recommendation – High-Quality Evidence).

### Cochlear implant in mastoid cavities

Patients with profound deafness require effective auditory rehabilitation and, when deafness is concomitant with chronic middle ear diseases, rehabilitation should be combined with disease eradication. While bone-anchored hearing aids can be positioned far from an open cavity or middle ear with secretion,^397^ CI is not placed in this same situation.

Many factors can hinder the use of CI in patients with open mastoid cavities, such as the presence of secretion, inflammatory tissue, residual cholesteatoma, patent eustachian tube, stenosis of the EAC, or extensive meatoplasty, which can lead to exposure, infection, and extrusion of the CI. Numerous surgical techniques have been described to minimize complications. These procedures include maintaining the mastoid cavity open by covering the electrode with fascia or bone pate, overclosing the EAC, reconstructing the posterior bony canal wall, and performing a middle fossa craniotomy to access the cochlea.^177^, ^398^, ^399^, ^400^

In favor of the maintenance of an open canal wall down mastoid cavity after CI are the speed and simplicity of the procedure, improved postoperative visualization, the ability to clean the cavity, and reduced risk of postoperative infections, breakdown of the EAC closure, development of residual cholesteatomas (due to cavity control), mucocele, and complications related to the fat donor site.^401^, ^402^ However, maintaining an open cavity is not without risk. Electrode extrusion may occur with cavity cleaning, in addition to being an open area susceptible to contamination from the external environment.^403^

To mitigate the risk of extrusion, some authors emphasize the elevation of the cavity’s fibroepithelial lining, minimizing the risk of leaving residual squamous tissue while providing adequate soft tissue coverage of the electrode.^404^, ^405^ Others describe additional electrode coverage using combinations of bone pate, fibrin glue, bone cement, perichondrium, bone matrix, cartilage, abdominal fat, and local musculoperiosteal flaps.^400^, ^406^, ^407^ Furthermore, to prevent the electrode array from looping within the open cavity, the implant can be placed more posteriorly than normal, creating gutter or zigzag grooves in the cortex of the temporal bone.^406^, ^407^ Olgun et al.^400^ described creating a tunnel underneath the facial nerve to the sinus tympani for passage, protection, and tethering of the electrode.

Subtotal petrosectomy was first described by Rambo in 1958^408^ and later modified and popularized by Fisch in 1965.^409^ The main steps of the procedure are: (1) Blind sac closure of the EAC; (2) Exenteration of the middle ear and mastoid, including perisigmoid, perilabyrinthine, perifacial, and hypotympanic cells; (3) Removal of the middle ear epithelium and mucosa; (4) Closure of the tympanic orifice of the eustachian tube; and (5) Obliteration of the cavity with abdominal fat.^410^

In 1998, Issing et al.^403^ and Bendet et al.^411^ proposed subtotal petrosectomy as the technique of choice for CI in ears affected by chronic otitis media. Subtotal petrosectomy allows complete isolation of the surgical cavity from the external environment, reducing the risk of postoperative infection, CSF leakage, and meningitis.^412^, ^413^ Furthermore, improved view and illumination of the surgical cavity provide excellent control and visualization of all available landmarks.^414^, ^415^ Properly performed subtotal petrosectomy results in a “safe and dry” cavity that is optimal for CI positioning.^416^ The wide angle of approach to the RW area, obtained by removing the posterosuperior wall of the EAC, facilitates electrode insertion.

Mastoid obliteration options include abdominal fat grafts, hydroxyapatite cement, and vascularized pedicled muscle flaps, including the temporalis muscle and even free flaps.^400^, ^404^, ^417^, ^418^ Abdominal fat is widely used due to its easy accessibility and reduced risk of necrosis and infection compared with muscle.^419^ However, creating a new surgical site introduces new risks, including hematoma and infection.^402^ Although large preoperative meatoplasties may discourage EAC overclosure due to the risk of postoperative breakdown, a meta-analysis has shown that EAC overclosure leads to fewer complications than maintenance of a canal wall down mastoid cavity with CI.^418^

Some algorithms have been developed for the management of CI in chronically infected ears.^401^, ^420^, ^421^ In order to reduce the risk of meningitis resulting from the insertion of an electrode into a potentially contaminated field^415^, ^422^ and residual cholesteatoma,^423^ CI has already been performed at different stages after surgery, ranging from concomitantly with surgery for removal of the disease up to 1-year later.^417^, ^421^, ^424^, ^425^ Most authors prefer to perform a second surgery before placing the CI, according to a meta-analysis that showed that 42.6% of cases with cholesteatoma were operated on more than once, while only 20.1% of cases were operated on in a single stage.^426^ However, another meta-analysis compared complication rates between patients who had the CI performed simultaneously with EAC overclosure and those who had the CI placed later, and there was no difference.^418^ Furthermore, depending on the case, especially in children, performing CI in a second stage can cause a significant delay in the hearing rehabilitation process.

In the presence of radical cavity or chronic otitis media with cholesteatoma, despite all efforts to meticulously remove all squamous epithelium, residual cholesteatoma may occur even many years after surgery.^411^, ^413^, ^415^ Therefore, prolonged follow-up of these patients is required. One of the protocols used includes monitoring patients with high-resolution CT at 1, 3, 5, and 10-years for the presence of cholesteatoma.^414^ The radiologic interface created by the fat in the cavity increases the diagnostic efficacy of high-resolution CT for residual cholesteatoma.^415^ Diffusion-weighted MRI, considered the gold standard for residual cholesteatoma, cannot be performed in the presence of a CI due to the enormous artifacts produced by this specific sequence. However, new generation implants allow the magnet to be removed and repositioned under local anesthesia immediately before and after MRI. This procedure can be performed once after 3-years. MRI-induced artifacts produced by a CI may not allow checking for cholesteatoma recurrence: even if tailored magnet positioning makes it feasible to monitor specific intracranial structures, its role in cholesteatoma detection is still debated.^401^, ^427^

Given the concerns about cholesteatoma development after EAC overclosure, some authors overclose the EAC without mastoid obliteration to allow for postoperative imaging to assess the modified middle ear for possible cholesteatoma, providing easier access in a revision.^398^, ^428^ However, postoperative imaging in unobliterated overclosed cavities shows very little air and mostly soft tissue in the mastoid cavities, suggesting the limited utility of postoperative imaging to assess for possible cholesteatoma.^428^ In addition, there are case reports of complications secondary to retrograde contamination of the middle ear through a patent eustachian tube.^429^, ^430^ Thus, closure of the eustachian tube is indicated. There are several techniques involving scarring the orifice mucosa with bipolar coagulation, stripping the mucosa, and/or obliterating with bone wax, muscle, cartilage, bone pate, myofascial grafts, perichondrium, periosteum, and/or fibrin.^415^, ^419^, ^425^

Farinetti et al.^431^ analyzed CI complications in 403 adults and children and found no significant difference in the rates of major complications between adults and children, in agreement with 2 other studies.^418^, ^432^ In a multicenter case series, with a mean follow-up of 44-months, only 12.7% of patients undergoing subtotal petrosectomy experienced complications.^433^ There are no significant differences in the rates of complications, reoperations, or device failures between the pediatric and adult populations.^426^ An additional, non-negligible benefit provided by subtotal petrosectomy is quality of life improvement in terms of clinical monitoring and daily living (swimming, vertigo induced by changes in pressure or temperature).^433^

While most mastoid cavities are either maintained or overclosed in conjunction with CI, 2 other surgical techniques have been described: reconstruction of the posterior wall of the EAC and the middle fossa approach.^176^, ^177^, ^407^, ^434^^,^^435^ Regarding the reconstruction of the EAC, Ito et al.^436^ reported a case of malformation of the inner ear and facial recess in which the posterior canal wall was recreated with a temporal bone plate and bone pate and reinforced with fibrin glue and a vascularized pedicle of temporal muscle. Tamura et al.^435^ reported on 3 patients who had posterior canal wall reconstruction with bone plates obtained from the mastoid cortex, followed by CI. However, there is always a risk that patients will develop complications such as postauricular incision dehiscence and development of residual cholesteatoma, but few reports are available in the literature. The middle fossa approach may be another option, but it requires craniotomy with retraction of the temporal lobe and also risks damage to the facial nerve.

#### Recommendations

LXXI Subtotal petrosectomy is more advantageous compared with alternatives such as mastoidectomy without EAC overclosure for placing the CI. It eliminates the need for routine monitoring of the open cavity and reduces the risks of infection and electrode extrusion. (Moderate Recommendation – Low-Quality Evidence).
LXXII In the case of profound hearing loss caused by cholesteatoma, subtotal petrosectomy should preferably be performed first, and CI in a second stage. (Moderate Recommendation – Low-Quality Evidence).
LXXIII The presence of a CI does not allow MRI to be performed for cholesteatoma recurrence, and CT is recommended for patient follow-up. (Strong Recommendation – Low-Quality Evidence).

### Cochlear implant and tinnitus

Tinnitus is a common symptom in CI candidates, with a prevalence ranging from 66% to 86%,^437^ and it can be disabling in some cases.^438^ A meta-analysis and systematic review demonstrated that CIs lead to improvements in tinnitus. Tinnitus Handicap Inventory (THI) scores significantly reduced by more than 50% after surgery in patients with different types of hearing loss and at different follow-up times.^439^

#### Recommendations

LXXIV In patients with profound sensorineural hearing loss and tinnitus, CI can be used as an option for auditory rehabilitation and tinnitus improvement. (Moderate Recommendation – High-Quality Evidence).

### Cochlear implant in patients with neurofibromatosis type 2 and vestibular schwannoma

CI has been indicated as a rehabilitation method for hearing loss associated with sporadic Vestibular Schwannoma (VS) or Neurofibromatosis type 2 (NF2) resulting from the disease itself or treatment.^440^, ^441^, ^442^, ^443^, ^444^ It has been indicated for unilateral and bilateral hearing loss, especially when associated with tinnitus.^440^, ^445^, ^446^, ^447^ One of the main arguments against CI in patients with VS is the impossibility of follow-up with MRI to assess the IAC postoperatively. Artifact reduction techniques have progressed and allowed good visualization of even the membranous labyrinth of CI users.^448^

Hearing rehabilitation in NF2, or sporadic VS in the only hearing ear, is a greater challenge due to the threat of bilateral hearing loss, bilateral hearing loss, from either the natural course of the disease or treatment, often at a young age. As a result, the traditional approach has been to wait for hearing loss to occur before surgical resection to preserve hearing function for as long as possible. However, the resulting larger tumor confers a greater risk at surgery, including facial nerve palsy and intracranial complications, in addition to loss of candidacy for hearing preservation surgery.^449^ A move toward earlier intervention in smaller tumors, with the goal of cochlear nerve preservation for CI, may reduce these additional surgical risks. In the case of NF2, a successful unilateral CI may also impact decision-making for a contralateral VS, with surgical treatment in an ear with residual hearing being considered in the knowledge that the patient will continue to function from an audiologic perspective.^450^

Most patients with VS experience a decline in hearing in the affected ear, regardless of whether observation, radiation, or surgery is selected as the primary treatment modality. Unilateral deafness has commonly been accepted as a consequence of disease progression or intervention in sporadic tumors. Rehabilitation options have previously been limited to contralateral rerouting of hearing aids or bone conduction devices (active or passive), with no attempt to provide auditory input to the affected ear.^451^ However, studies of CI in non-tumor-related unilateral deafness have shown significant benefits in spatial perception, sound localization, speech intelligibility, and quality of life in patients with a contralateral hearing ear.^452^ Some patients have difficulty integrating the signal from their CI with their natural hearing. However, this is not universal, and trends show improvement over time. CI is a feasible method of hearing rehabilitation in selected cases of sporadic VS regardless of the hearing status of the contralateral ear and is being increasingly used.^453^

CI is considered an option in cases where there is anatomic and functional preservation of the cochlear nerve, since CI outcomes may be superior to those of ABI with lower morbidity rates.^445^, ^454^ EABR has been used for intraoperative assessment of cochlear nerve function after VS resection, assisting in the selection of patients who may benefit from a CI.^447^, ^454^ The electrode array is placed in the RW niche or within the cochlea, with the recording electrodes being placed on the scalp. Although it can be used during hearing preservation surgery, sound stimulation with recording electrodes optimally either on the cochlear nerve (measuring the cochlear nerve action potential) or on the brainstem in the foramen of Luschka (measuring the dorsal cochlear nucleus action potential) can cause substantial artifact, making these recording sites less useful. However, a robust EABR has been correlated with a good post-CI auditory outcome.^455^, ^456^

Preservation of the cochlear nerve and improved CI response occur more frequently in tumors up to 2 cm.^457^, ^458^, ^459^ CI has been used in patients with VS treated with observation, with imaging follow-up and growth stability over the last 2–3 years,^458^, ^460^, ^461^ and in patients treated with microsurgery, even with a hearing preservation technique that evolves with hearing loss.^446^, ^458^ In patients undergoing translabyrinthine resection, cochlear ossification has been reported postoperatively, with the percentage of completely patent cochleae decreasing from 75% to 38% in 6–12 months after surgery. When performing CI in a second stage, placement of a dummy electrode in the cochlea is recommended to maintain cochlear patency.^462^, ^463^, ^464^ Furthermore, there is evidence that spiral ganglion cells can deteriorate after surgery, leading to poorer hearing outcomes.^465^

West et al.,^446^ in a review of 86 patients undergoing tumor surgery (via middle fossa, retrolabyrinthine, retrosigmoid, or translabyrinthine approach), found no difference in CI outcomes between patients with sporadic VS or NF2, with mean discrimination scores of moderate-to-high performances and only a few cases where there was no response. The authors emphasized the difficulty of comparing different studies since the tests used for audiologic evaluation were different.

Iannacone et al.,^458^ in a review study, recommended that CI be performed simultaneously to translabyrinthine resection of VS, based on reports of up to 40% of cochlear ossification at 1 year after tumor resection. Conversely, Wick et al.^466^ found no significant difference between simultaneous and sequential CI in a literature review of 93 patients.

Arnoldner et al.^443^ established hearing loss as ≤50% monosyllable recognition at 80 dB, Deep et al.^457^ as mean pure-tone thresholds >70 dB, and Mukherjee et al.^462^ as thresholds >90 dB. Sorrentino et al.^441^ considered CI indication for patients with moderate-to-severe hearing loss in the contralateral ear and an indication for translabyrinthine surgery in the ipsilateral ear. This prospective study considered the presence of positive intraoperative EABRs after translabyrinthine resection of sporadic VS an indication of cochlear nerve function preservation to allow simultaneous CI. Of 17 patients, 10 received a CI.^443^ They also observed improved postoperative responses in patients who had residual speech recognition preoperatively, ranging from 20% to 65% in 9 patients 6-months after surgery. One patient had no response.

Bartindale et al.,^467^ in a systematic review of 45 studies including 15 patients with sporadic VS, found that the mean speech discrimination score improved from 30% to 56.4%, with better results in patients who showed poorer discrimination preoperatively. Tumor location and size, duration of deafness before CI, and type of CI (sequential or concomitant) were not associated with postoperative hearing outcomes.

Deep et al.^457^ retrospectively evaluated 24 patients with NF2 treated with microsurgery, irradiation, or observation who received a unilateral CI. Mean patient follow-up was 4-years. The rate of open-set speech perception was 42%, with better results in tumors 1.5 cm or less.

Sanna et al.^447^ retrospectively evaluated 41 patients (33 with sporadic VS and 8 with NF2) undergoing unilateral resection of tumors 2 cm or less via the translabyrinthine approach with simultaneous CI. One year after surgery, 48.8% of patients still used their CI, with a sentence recognition rate of 60.7%. Those who stopped using the CI had a sentence recognition rate of 15.3%. There was no statistically significant difference between patients with sporadic VS and NF2. Unlike the study by Bartindale et al.,^467^ better preoperative audiologic test results had a positive correlation with postoperative results, which may indicate better preservation of neural elements.

Longino et al.^461^ retrospectively evaluated 7 patients with sporadic VS treated with observation and showed tumor growth stability. Consonant-nucleus-consonant word scores improved from 6% to 55% and AzBio scores in quiet improved from 9% to 56% 6-months after CI, and these results were maintained at 12-months.

Sorrentino et al.^441^ evaluated 8 patients with sporadic VS and 9 with NF2 24-months after CI; one of the patients received a bilateral CI. CI was simultaneous in 11 ears and sequential in 7 and occurred between 1- and 3-months after VS resection. The results were better in patients with sporadic VS and worse in those with better contralateral hearing, while preoperative hearing had no effect on the implanted side. The mean word recognition score was 40%.

Regarding tinnitus, West et al.^446^ retrospectively evaluated 22 patients, 21 with sporadic VS and 1 with NF2. Nineteen patients underwent tumor resection, 1 received radiotherapy, and 2 were treated with observation. Sixteen patients had unilateral deafness. The THI was administered to 17 patients both preoperatively and postoperatively, with a significant score reduction in 76%, no changes in 18%, and increase in 6%. Conway et al.^445^ prospectively evaluated 10 patients with sporadic VS who underwent translabyrinthine resection with simultaneous CI and found a significant reduction in THI scores (from 41.3 to 23.3) 3-months after surgery. Tian et al.^468^ reviewed 33 patients who received radiotherapy and reported improved speech discrimination and reduced tinnitus in 70% of cases after CI surgery.

There is concern about whether imaging can confirm the effectiveness of tumor resection, detect recurrences, or control for residual lesions. However, satisfactory evaluation has been obtained. Novel bipolar magnets align with the magnetic field and reduce the likelihood of CI displacement and pain during imaging. Changes in CI positioning have allowed MRI to be performed with 1.5-T and 3.0-T scanners without causing many artifacts in the IAC region.^469^, ^470^ As for the surgical technique, positioning the receiver 8–9 cm from the EAC at an angle of 90 ° or 160 ° in relation to a line that passes through the nasion (anterior portion of the frontonasal suture) and the EAC allows adequate visualization of the IAC and the cerebellopontine angle.^469^, ^470^

Long-term post-CI hearing outcomes in VS are scarce in the literature. Neff et al.^471^ reported that hearing performance did not significantly deteriorate over an extended postoperative follow-up, with a maximum follow-up of 93-months. Given the less predictable growth pattern of NF2-associated VS, the proportion of patients with NF2 who will potentially lose benefit from CI caused by tumor regrowth and will require ABI for hearing rehabilitation remains to be determined.^457^ Overall outcomes for CI in patients with VS show favorable results with observed or irradiated tumors, sporadic and NF2-related, but outcomes after tumor resection are more variable with a greater chance of no benefit.^450^, ^457^ Despite poorer outcomes in patients with VS than seen in the general CI population, a CI should always be considered in a stable or resected tumor with cochlear nerve preservation because it will almost always outperform an ABI.^471^, ^472^

As highlighted by Bartindale et al.;^467^ the priorities of treatment in VS are in order of importance to preserve life, facial function, and hearing, as well as to minimize other complications. Hearing preservation should remain a key factor in decision-making for patients with VS. Schlacter et al.,^472^ in their meta-analysis, reported that 82% of patients had improved speech perception scores after CI. The same proportion were regular daily users (defined as more than 8 h per day). Likewise, Smith et al.^473^ reported that 84.9% of recipients remained regular daily CI users after a median follow-up of 3.6 years. Although uncertainty remains regarding long-term hearing outcomes, CI offers significantly better results than ABI.^450^, ^467^, ^471^ Neither the primary treatment modality nor the cause of the tumor (NF2-related or sporadic) appears to have a significant effect on outcome. CI in VS serves to offer patients, with a functioning cochlear nerve, the probability of open-set speech discrimination with a consequent positive impact on quality of life.

#### Recommendations

LXXV CI may be indicated in patients with hearing loss due to sporadic VS undergoing surgery or radiotherapy when there is anatomic and functional preservation of the cochlear nerve. In cases of unilateral deafness, especially in cases of disabling tinnitus, the risks and benefits should be considered, as well as the additional care required for postoperative imaging follow-up. (Moderate Recommendation – Moderate-Quality Evidence).
LXXVI Patients with NF2 may receive a CI, even without tumor resection or with partial resection (if it is neurologically safe), because this approach can increase the chances of cochlear nerve function preservation. (Moderate Recommendation – Moderate-Quality Evidence).
LXXVII In patients undergoing translabyrinthine tumor resection, if CI is chosen for hearing rehabilitation, it should preferably be performed simultaneously to tumor resection. (Moderate Recommendation – Moderate-Quality Evidence).
LXXVIII Patients with intracochlear or intralabyrinthine VS can receive a CI even without tumor resection due to the low risk of growth of schwannomas located in these regions. (Moderate Recommendation – Low-Quality Evidence).

### Cochlear implant complications

There are complications inherent in mastoidectomy and posterior tympanotomy, presence of an internal device in the patient (foreign body), and failure of the implanted device. The rate of these complications has decreased over time due to improvements in surgical techniques and technological developments, currently standing at approximately 9%.^431^ It is important that patients, family members, and health professionals be aware of potential complications. In addition to the consequences on patients’ health, there is also the economic impact on the public health system and private health insurers.

In 2009, Hansen et al.^432^ defined complications as minor or major (Table 5). The authors also divided them into medical and surgical, adult and pediatric, perioperative (occurring up to 24 h after the end of surgery), early postoperative (within 1 week of surgery), and late postoperative (beyond 1 week of surgery). Complication rates reported in the literature are 11.8% for minor complications and 3.2% for major complications.

**Table 5:** Minor and major complications of cochlear implant surgery.

Major complications	Minor complications
Surgical wound infection with exposure of the internal component	Superficial surgical site infection
Permanent facial paralysis	Temporary facial paralysis
Persistent vertigo	Mild dizziness
CSF leak	Gusher or Ooze
Electrode migration or misplacement	Tympanic membrane perforation
Internal device failure	Fenestration of the posterior wall of the EAC
Meningitis	Dysgeusia
Cholesteatoma	Facial nerve stimulation
Persistent otalgia	Tinnitus

CSF, Cerebrospinal Fluid; EAC, External Auditory Canal.

Major complications are defined as those requiring revision surgery or hospitalization for medical treatment: 1) Serious medical problem (e.g., meningitis); 2) Complication requiring additional, major surgical intervention (e.g., cholesteatoma or explantation); 3) Any degree of permanent disability (e.g., permanent facial paralysis).

Minor complications are defined as those requiring conservative treatment or minimally invasive surgery, such as ventilation tube placement, that do not meet the criteria for major complications.

#### Infection

It is the most common cause of postoperative complications, with rates ranging from 1.7% to 12%.^474^, ^475^

#### Middle ear infections

Middle ear infections, such as AOM, serous otitis media, and mastoiditis, are more common in children, especially in the first 2-years of life. In CI users, they may lead to CI extrusion or failure and meningitis. The incidence of meningitis in the general population is 0.5–5.0 cases per 100,000 patient-years.^221^ Implanted patients have a higher risk of central nervous system infections than the general population (30 times);^227^ therefore, they should be vaccinated for prevention. The electrode array is a route for spreading infection from the middle ear to the cochlea (ST that communicates with the cochlear aqueduct and subarachnoid space). The main agent involved in cases of CI-related meningitis is pneumococcus,^231^ which is why vaccination is very important, as discussed earlier.

AOM within the first 2-weeks of surgery can cause severe cochlear damage, responsible for deterioration of residual hearing, vestibular disorders, and cochlear ossification.^475^ The risk of meningitis can increase by up to 50% in these cases.^227^ Some rehabilitation centers perform ventilation tube placement and/or adenoidectomy before CI surgery in children with a history of recurrent AOM or serous otitis media.^227^, ^475^, ^476^, ^477^

Risk factors for infections: (1) Age at CI surgery less than 2-years and more than 65-years; (2) Immunosuppression; (3) History of spontaneous or traumatic CSF leak; (4) Presence of neurosurgical prostheses; and (4) History of meningitis.^475^ Other risk factors are more directly related to the ear: (5) Inner ear malformations and (6) History of ear surgery (stapedotomy).^478^

#### Skin infections

Surgical wound complications may occur and are potentially devastating. They may compromise the patient’s health and hearing outcomes. As a relatively low-risk outpatient procedure, the rate of medical complications, such as wound compromise, among CI recipients has been estimated to be 2.66%–4%.^479^, ^480^ In rare situations, CI wound complications may require explantation of the device.^475^, ^481^ These complications may occur immediately or even years after surgery and include infection, wound collapse, hematoma, or seroma formation.

A fundamental understanding of the patient’s underlying physiology and health is essential for decision-making and counseling for any type of surgery. Chronic medical conditions, patient age, and use of anticoagulants are all factors that influence health and potentially have an impact on surgical outcomes. There is a lack of quantitative meta-analysis data to assess for risk and association of patient-level factors with wound complications and a clearer understanding of these factors and the relative risks for wound complications would be valuable for patient counseling and surgical decision-making.

Superficial infections at the incision site or flap are common. They should be promptly treated with antibiotic therapy and dressings on an outpatient basis. If exposure of the internal component occurs, explantation is indicated. Bi et al.^482^ suggested classification and management of late skin infections in children according to severity: type (A) – flap seroma or hematoma around the internal component; type (B) – skin flap infection or necrosis with development of granulation tissue over the wound, without CI exposure; and type (C) – skin flap rupture with CI exposure and extrusion.

The mechanisms underlying these surgical wound complications are poorly understood. Perioperative management and surgical techniques can influence CI wound healing.^483^ Recurrent AOM may play a role in seeding a wound bed and predisposing to pediatric CI wound infections.^484^, ^485^ Scalp thickness is a significant patient-level factor that can also influence adult and pediatric wound complications. Foreign body reactions are also a proposed mechanism for wound breakdown and may be related to a reaction to the CI itself or to the suture material used to secure the CI.^486^ Olsen et al.^479^ found a 10% surgical wound infection rate in adult patients and that *Staphylococcus aureus*, often with biofilm formation, was the main pathogen involved in these complications.

### Cholesteatoma

Patients rarely develop cholesteatoma after CI. It may result from damage to the tympanic membrane and/or EAC during surgery. Treatment consists of CI explantation at the site of infection and biofilm formation, in addition to conversion to a canal wall down mastoid cavity and excision of the entire lesion. It is important to maintain only the electrode array inside the cochlea and remove the rest of the device to preserve cochlear patency and avoid fibrosis. CI reimplantation should be performed as soon as possible, after infection control, combined with subtotal petrosectomy.^487^

### Peripheral facial palsy

Peripheral Facial Palsy (PFP) is one of the worst complications that can occur in ear surgery. When opening the mastoid to resect tumors or treat infections, although undesirable, PFP can be an iatrogenic complication of the procedure. However, in CI surgery, in patients with normal anatomy, PFP may be disastrous. The incidence of PFP is low (0.4% to 0.7%).^488^, ^489^

Continuous IFNM has been used for CI surgery.^490^ There is scarce literature on the effectiveness of IFNM in reducing the risk of postoperative facial paralysis in CI surgery. Only 2 articles have briefly discussed the topic, but the results are conflicting.^489^, ^491^ Fayad et al.^489^ reported that there is no relationship between the use of IFNM and delayed facial nerve palsy. However, Thom et al.^491^ observed a 4.5-fold increased risk of delayed postoperative facial nerve palsy in cases where IFNM was not used.

Although some studies imply a weak association between IFNM use and postoperative facial nerve function and, therefore, fail to validate the standardized use of IFNM in CI surgery, the merit of using a monitoring system as an additional precautionary device to prevent further nerve injury should be recognized. IFNM is also useful in training surgeons and enhancing their skills under the guidance of experienced surgeons.^492^ Furthermore, the use of IFNM is strongly encouraged in cases of otitis media, mastoiditis, or abnormal inner ear morphology due to poor visibility and low predictability of facial nerve location in these patients.^298^, ^476^

Intraoperative factors, including mechanical trauma and thermal injury, can lead to progressive inflammation, neural edema, and ischemia, which, in turn, can result in immediate-onset or early-onset delayed palsy. Thermal injury may occur due to inadequate irrigation or excessively vigorous drilling of the facial recess. In several previous studies, thermal injury has also been reported when the “neck” of the drill is accidentally placed against the facial nerve during drilling to identify the RW membrane or cochleostomy.^491^, ^493^ The use of copious irrigation may reduce the possibility of burr heating and thus reduce the risk of thermal injury and neural edema. Although necessary, the use of IFNM does not replace the surgeon's knowledge of anatomy and proper technique.

Identification of the facial nerve is essential to avoid severe injuries and complications. Preoperative imaging, especially in patients with malformations, can prevent complications. If immediate PFP is detected, it is essential to re-approach the mastoid and decompress the facial nerve as quickly as possible. The prognosis is more favorable when there is only edema. However, the occurrence of damage to the facial nerve fibers increases the risk of permanent paralysis.

#### Delayed facial palsy

In some cases, delayed PFP may occur (a few hours or even days after the procedure). The disparity in timing and clinical course between immediate and delayed facial palsy suggests that the mechanisms of injury are different. Intraoperative factors, including mechanical trauma and thermal injury, can lead to progressive inflammation, neural edema, and ischemia resulting in paralysis a few hours after the end of surgery.

The pathophysiology of PFP a few days after surgery is poorly understood. Studies have shown the onset of paralysis 8 days after the procedure.^494^ Several theories have been proposed, but reactivation of a latent herpes simplex or zoster virus present in the geniculate ganglion seems the most likely cause. Reactivation may occur after manipulation, heat transfer, damage to the chorda tympani nerve or other sensory branches of the facial nerve. Major increases in herpes simplex types 1 and 2 and varicella zoster Immunoglobulin (Ig) M and/or IgG levels have been demonstrated in patients with delayed facial palsy after stapedectomy and VS surgery.^488^

Treatment for PFP is similar to that for other causes. The use of high-dose corticosteroids is indicated. However, the benefit of combined corticosteroid and antiviral therapy remains controversial, with most randomized controlled trials and meta-analyses demonstrating no advantage over corticosteroids as monotherapy. Approximately 0.1% of patients with delayed symptoms have incomplete recovery.^494^

### Facial nerve stimulation

The rate of facial nerve stimulation ranges from 0.9% to 14.9% in CI users. It is usually associated with otosclerosis (most common), cochlear malformation, and temporal bone fracture. It may also result from extrusion or partial insertion of the electrodes and direct stimulation of the facial nerve, mainly in the mastoid portion. The most common symptoms are paresthesia, visible facial twitching, and pain.^357^

Facial nerve stimulation during CI use is more common in patients with advanced otosclerosis, reaching up to 17% of cases in some studies.^358^, ^495^ It may be a minor or major complication, often resolved by reprogramming or deactivating part of the electrodes. However, when many electrodes need to be deactivated, CI performance can be negatively impacted. In some cases, explantation may be necessary.

The risk of facial nerve stimulation in advanced otosclerosis is lower with perimodiolar electrodes. Migration of the electrode array into the IAC may also occur. In this case, imaging can be useful in making the diagnosis and assessing the need for electrode repositioning. Patients with cochlear malformations, such as CC deformity, IP-I, and IP-III, are at increased risk of electrode migration into the IAC. Other unpleasant symptoms that may develop after CI in far advanced otosclerosis are tinnitus, dizziness, and headache.

### Electrode array misplacement

It is one of the most common errors made by inexperienced surgeons. Incomplete electrode insertion occurs in up to 2% of patients with normal cochlear anatomy, being more prevalent in straight banded arrays than in perimodiolar arrays.^496^

For optimal hearing outcome, the electrode array must be inserted into the ST. The best way to ensure that the electrodes will be positioned at this location is to insert them via the RW. Sometimes, the RW can be confused with hypotympanic cells that are inadvertently opened, and when attempting to insert the CI, the internal component may break. To avoid this type of error, in addition to properly identifying the RW, the pyramidal eminence and stapes should be located. Drilling of the superior lip of the RW niche exposes the window more adequately. Another way to confirm the proper location of the RW is to move the handle of the malleus and check whether the RW membrane also moves.

### Internal device failure

Some authors consider it a major complication because the patient requires rehospitalization and revision surgery to replace the device. It occurs in approximately 2%–4% of cases. It is more common in children than in adults due to trauma. Over the years, failure rates have decreased due to improved device quality and increased strength of the internal component. When a device failure is confirmed, a mastoid CT should be performed to check whether the electrodes are properly positioned inside the cochlea and reimplantation should be performed as soon as possible.^195^

### Magnetic resonance imaging and its impact on hearing implants

The use of MRI has been expanded particularly to include non-neurologic applications such as the heart, abdomen, pelvis, and musculoskeletal system. The use of MRI is expanding at a rate of approximately 20% per year.^497^

Pain is the most common complication in CI users undergoing MRI and can occur in up to 70% of patients, but only in about 6%–18% it prevents scanning from being completed.^498^ Other significant complications are magnet dislocation (0.6%–15%),^488^ depolarization, and polarity reversal.^497^ Artifacts caused by CI remain a problem but can be reduced by using specific sequences. Noise perception during MRI has rarely been described in CI users, but there are reports of interruption of the scanning procedure, in a 1.5-T scanner, due to pain and noise perception.^499^

The induction of electric currents in response to rapidly changing electromagnetic fields and radiofrequency pulse emission could lead to heating of muscle tissue, skin damage, and internal device malfunction. However, studies of CIs have not yet demonstrated relevant temperature increases that would pose a risk to the surrounding tissue.^500^, ^501^ There is no correlation between pain and MRI duration or head positioning.^502^, ^503^ In the studies evaluated, from 2.25% to 17% of MRI scans had to be stopped because of patient pain.^498^, ^502^, ^504^ Transient erythema or pressure ulcers may also occur, most likely due to patient wearing a tight bandage around the head.^503^ Other studies and case reports have described severe pain often associated with magnet displacement, but which could also result from wearing a tight headband.^505^, ^506^

Displacement of the internal magnet caused by the MRI magnetic field has been considered a serious risk and even a contraindication to CI in the past.^507^ But CI has evolved over the years. Currently, there are devices compatible with MRI up to 3.0 T. However, some studies have shown CI-related impairment and complications, especially in earlier implant models. Tam et al.,^508^ in a prospective study, found a magnet displacement rate of 3.5% for MRI scanning in CI or ABI users using a 1.5-T scanner. Loth et al.^509^ reported a magnet dislocation rate of 7% in their retrospective study of 711 patients. Demagnetization and magnet polarity reversal are rare and poorly reported events.^510^, ^511^ Magnet polarity reversal or demagnetization during MRI is a relatively rare complication that may result in reduced retention of the external speech processor.

A study conducted in a cadaveric model showed that a pressure head bandage over the CI reduces the risk of magnet dislocation during MRI.^512^ However, a head bandage does not appear to completely prevent magnet dislocation.^513^ Hassepass et al.,^497^ in a retrospective study with an observation period of 13-years, found 23 cases of magnet dislocation in CI users. Ten of these cases occurred despite the use of a bandage without a counter-pressure element. This suggests that either the bandage does not reliably reduce dislocation, or the dressing technique needs to be improved. Finally, it does not appear to make a significant difference whether the preventative head bandage is applied by a trained radiologist or an otolaryngologist.^514^

In the available prospective and retrospective studies, the use of head bandages has not been reliably documented in all patients and the method of implementation is variable.^497^, ^502^, ^504^, ^508^ It is important to always follow the manufacturer’s recommendations for limiting the field of MRI scanning. In the study by Kim et al.,^498^ 2 of the 18 study patients did not receive a head bandage and were also scanned at 3.0 T, which contradicts the manufacturer’s recommendations for the hearing implants used.

Most CIs are compatible with 1.5 T field strength. Devices that support field strengths of up to 3.0 T are now available on the market.^515^ The manufacturer’s recommendations should be followed to reduce the risk of complications, although they can occur even when the guidelines are followed.

To ensure that implants at risk can also undergo MRI scanning, it is necessary to provide specialized care for CI users during the imaging procedure. This could be regulated by standard operating procedures, of which the procedure described here could be a variant. This would help ensure compliance with the manufacturer’s precautions and the MRI parameters required for each implant type.

Additional studies would be useful to consolidate MRI safety conclusions for currently implanted CIs and to increase the safety of imaging older models. This may also help address the current refusal of CI users by imaging centers.

### Migration of the electrode array in the cochlea

Electrode positioning complications represent a significant proportion of perioperative CI complications and compromise patients’ hearing gain. Careful surgical planning and appropriate preoperative and intraoperative imaging can reduce the risk and impact of electrode positioning complications.

In 2003, Eshraghi et al.^516^ developed a grading system for intracochlear trauma caused by insertion of 3 different types of perimodiolar electrodes in cadavers (Table 6).

**Table 6:** Grading system for intracochlear trauma caused by perimodiolar electrodes. Adapted from Eshraghi et al.^516^

Grade	Damage
0 (zero)	No trauma (atraumatic insertion)
1	Mild elevation of basilar membrane, without rupture
2	Rupture of basilar membrane
3	Scalar translocation of the electrode
4	Fracture of the modiolus wall or tear of the stria vascularis

In a non-ossified cochleae with normal anatomy, the electrodes should remain fully in the ST after insertion. Scalar Translocation (STL) of the electrode array occurs when the electrode migrates from the ST to the scala vestibuli or media, piercing the cochlear partition. It reduces the patient’s residual hearing, in addition to leading to a more unfavorable position of the electrode array for stimulation of the cochlear nerve. Straight electrodes have a significantly lower risk of STL than perimodiolar electrodes (7% vs. 43%), even when only RW insertion is considered (2% vs. 22%).^517^

Electrode tip fold-over or rollover is another complication of CI insertion. It is uncommon, less than 2% of surgeries, being more frequent in perimodiolar electrodes. It occurs because the electrode tip folds during insertion, which may not be noticed by the surgeon. Typically, intraoperative neurotelemetry is normal. It can be corrected before waking the patient if identified by intraoperative imaging or in revision surgery or if identified postoperatively if patients experience vertigo, unfavorable hearing performance, or tinnitus.^518^

### Dizziness

CI can have a significant impact on vestibular function. Multiple mechanisms that may lead to vestibular dysfunction during or after CI surgery have been proposed: (1) Direct trauma from electrode insertion; (2) Acute serous labyrinthitis due to cochleostomy; (3) Oreign body reaction with labyrinthitis; (4) Endolymphatic hydrops; and (5) Electrical stimulation from the implant itself.^77^ The occurrence of vestibular dysfunction after CI surgery has a very wide range as assessed by caloric testing and VEMP.^77^, ^519^ However, not all CI users experience postoperative dizziness.^519^, ^520^ Kluenter et al.^519^ reported different forms of dizziness after surgery. Given the increasing use of bilateral implants, it is important to quantify the effects of CI surgery on the vestibular system. This information is of great benefit to both the CI team and patients.

The reported incidence of vestibular dysfunction after CI surgery ranges from 39% to 74%, lasting from 2 days to 2 years.^521^ Only a few patients report significant postoperative symptoms. However, vestibular assessment is important in cases that will undergo unilateral surgery. If the chosen side has good vestibular function and the contralateral side has vestibular hypofunction, there is a high risk of causing bilateral vestibular hypofunction, which may lead to loss of balance with great limitation in daily activities. In these cases, it is preferable to choose the side with worse vestibular function.^522^

The onset of vertigo can occur immediately after surgery or as a delayed phenomenon. The electrode array impairs the physiologic function of the organ of Corti and adjacent labyrinthine structures.^523^ Other possible mechanisms include changes in vestibular receptors and effects on the central nervous system.^524^ Patients with profound hearing loss may have vestibular dysfunction and report vertigo before surgery. Children rarely experience long-term vertigo.^525^ However, vestibular dysfunction and balance impairment have been identified as important risk factors for CI failure in children.^526^ Older patients more frequently complain of vertigo after CI.^527^

The saccule is the most affected organ. CI surgery has a significant negative effect on caloric testing and VEMP in adults. The factors that most influence symptoms are advanced age and etiology of hearing loss.^92^ Younger patients may compensate better after vestibular dysfunction.^528^ Hearing preservation techniques and electrode insertion via the RW also appear to better preserve vestibular function.^529^ Therefore, although rare, the possible effects of CI surgery on the vestibular system should be communicated to patients before surgery.

### Meniere’s disease

Most patients with Meniere’s Disease (MD) will have different degrees of permanent hearing loss, and for 15%–38% of patients with unilateral MD, hearing loss will progress to severe sensorineural hearing loss.^530^ Bilateral MD rarely presents with both ears affected simultaneously. Most cases occur sequentially with initial unilateral symptoms progressing to bilateral disease.^531^ Management of this condition aims to minimize vestibular symptoms while preserving hearing as much as possible. Most patients are controlled by lifestyle modifications and medication use. If unsuccessful, intratympanic injections (gentamicin or corticosteroids), endolymphatic sac decompression, or vestibular neurectomy can be proposed, with these procedures entailing the risk of hearing loss. Surgical labyrinthectomy is an effective treatment for vertigo attacks, but this procedure forfeits residual hearing and may trigger cochlear ossification.^532^

CI has also been described for patients with MD, yielding favorable hearing outcomes.^533^ A systematic review evaluated 17 studies that showed CI safety and efficacy in 182 patients with MD.^534^ Despite the heterogeneity of studies, patients with DM had significant hearing improvements after CI and a complication rate comparable to that of CI users without MD. The results were similar to those in the general population of CI users. Only 3 patients (1.6%) had a decline in speech perception scores. Of the patients reporting postoperative vertigo, none had undergone simultaneous or delayed labyrinthectomy with CI.

However, patients with MD may pose a challenge postoperatively due to fluctuating performance that requires device reprogramming.^533^, ^535^ In patients with bilateral MD, the status of the contralateral vestibular system should be considered in CI, as surgery may result in additional vestibular damage. Patients with MD who have previously undergone labyrinthectomy may require updated imaging (MRI) to ensure the presence of a patent cochlear duct. Approximately one-third of patients undergoing labyrinthectomy develop cochlear ossification, which could preclude electrode array insertion.^535^, ^536^

#### Recommendations

LXXIX Patients with surgical wound infection should be evaluated and treated as quickly as possible with local dressings and broad-spectrum antibiotics to avoid CI exposure that will lead to explantation surgery. (Strong Recommendation – Moderate-Quality Evidence).
LXXX Patients with infections such as surgical wound dehiscence or cholesteatoma who require device replacement should undergo two-stage surgery, maintaining the electrode array inside the cochlea to avoid ossification. (Moderate Recommendation – Moderate-Quality Evidence).
LXXXI Middle ear infection should also be treated with antibiotics due to the risk of causing cochlear damage, ossification, meningitis, and implant failure. (Moderate Recommendation – Moderate-Quality Evidence).
LXXXII Although encouraged, the use of IFNM in CI surgery does not replace the surgeon’s need for knowledge of anatomy, being more relevant for patients with malformations. (Strong Recommendation – Low-Quality Evidence).
LXXXIII Immediate facial palsy can be caused by mechanical trauma or thermal injury to the facial nerve during surgery and requires immediate facial nerve decompression. (Strong Recommendation – Moderate-Quality Evidence).
LXXXIV Delayed facial palsy can be caused by inflammation, edema, or viral reactivation in the facial nerve and may be treated with corticosteroids. (Moderate Recommendation – Moderate-Quality Evidence).
LXXXV Facial nerve stimulation can cause paresthesia, facial twitching, and pain and may be resolved by reprogramming or deactivating part of the electrodes. (Moderate Recommendation – Moderate-Quality Evidence).
LXXXVI Internal device failure can be caused by trauma, infection, electrode migration, electrode misplacement, or issues related to electronic equipment. It requires rehospitalization and revision surgery to replace the device, which can be performed immediately after explantation of the failed internal component. (Moderate Recommendation – Moderate-Quality Evidence).

## Conclusion

CI is a safe device for auditory rehabilitation of patients with severe-to-profound hearing loss. In recent years, indications for unilateral hearing loss and vestibular schwannoma have been expanded, with encouraging results. However, for a successful surgery, commitment of family members and patients in the hearing rehabilitation process is essential.

## Funding

The authors have no financial relationships relevant to this article to disclose.

## Conflicts of interest

The authors declare no conflicts of interest.
